# Histone H3 Localizes to the Centromeric DNA in Budding Yeast

**DOI:** 10.1371/journal.pgen.1002739

**Published:** 2012-05-31

**Authors:** Berit Lochmann, Dmitri Ivanov

**Affiliations:** Friedrich Miescher Laboratory of the Max Planck Society, Tübingen, Germany; Duke University, United States of America

## Abstract

During cell division, segregation of sister chromatids to daughter cells is achieved by the poleward pulling force of microtubules, which attach to the chromatids by means of a multiprotein complex, the kinetochore. Kinetochores assemble at the centromeric DNA organized by specialized centromeric nucleosomes. In contrast to other eukaryotes, which typically have large repetitive centromeric regions, budding yeast CEN DNA is defined by a 125 bp sequence and assembles a single centromeric nucleosome. In budding yeast, as well as in other eukaryotes, the Cse4 histone variant (known in vertebrates as CENP-A) is believed to substitute for histone H3 at the centromeric nucleosome. However, the exact composition of the CEN nucleosome remains a subject of debate. We report the use of a novel ChIP approach to reveal the composition of the centromeric nucleosome and its localization on CEN DNA in budding yeast. Surprisingly, we observed a strong interaction of H3, as well as Cse4, H4, H2A, and H2B, but not histone chaperone Scm3 (HJURP in human) with the centromeric DNA. H3 localizes to centromeric DNA at all stages of the cell cycle. Using a sequential ChIP approach, we could demonstrate the co-occupancy of H3 and Cse4 at the CEN DNA. Our results favor a H3-Cse4 heterotypic octamer at the budding yeast centromere. Whether or not our model is correct, any future model will have to account for the stable association of histone H3 with the centromeric DNA.

## Author Summary

During cell division, replicated DNA molecules are pulled to daughter cells by microtubules, which originate at the spindle poles and attach to a multiprotein complex, the kinetochore. The kinetochore assembles at a special region of the chromosome, termed the centromere. The kinetochore is comprised of more than 50 different proteins whose precise functions are far from being fully understood. The kinetochore assembles on the foundation of a specialized centromeric nucleosome. A nucleosome is a complex of eight subunits, termed histones, which compacts the DNA by wrapping it around itself in 1.7 turns of a superhelix. The centromeric nucleosome is very special, and its stoichiometry and structure are a subject of intense debate. It is believed that the centromeric nucleosome is devoid of histone H3 and instead contains its variant, termed CENP-A in vertebrates or Cse4 in budding yeast. Here we report that in budding yeast both CENP-A and histone H3 localize to a small centromeric DNA fragment that, due to its size, cannot accommodate more than a single nucleosome. Our results necessitate a revision of what is known about the structure of the inner kinetochore and the role of CENP-A in its assembly.

## Introduction

During eukaryotic cell division sister chromatids, containing identical copies of genetic information, are pulled apart and driven towards opposite spindle poles by the microtubules of the mitotic spindle, which attach to the centromeric DNA sequences of the sisters via kinetochore protein complexes. It is imperative for proper chromosomal segregation that each chromosome assembles the kinetochore only at one site. The sites of kinetochore assembly are marked by specialized nucleosomes. Budding yeast represents the simplest case in which a single microtubule attaches to the so-called “point” kinetochore assembled around a single centromeric nucleosome. More complicated “regional” centromeres of most other eukaryotes are composed of arrays of specialized centromeric nucleosomes interspersed with conventional nucleosomes [1] and support the assembly of several microtubule attachment sites.

Centromeric nucleosomes were reported to have histone H3 substituted by a histone variant, CENP-A, called Cse4 in budding yeast [2]. It displays more than 60% similarity with the conventional histone H3 within the histone fold domain and has an additional N-terminal extension [3]. CENP-A has been demonstrated to co-purify with a subset of kinetochore proteins and is likely to provide interaction surfaces for kinetochore assembly [4], [5]. Recruitment of CENP-A to centromeric DNA requires the CENP-A targeting domain (CATD), comprised of loop1 and the α2-helix [6], [7], and is regulated by a number of other proteins [8]. One example is the non histone protein Scm3 (HJURP in human [9]), which is believed to be a histone chaperone required for recruitment of CENP-A to centromeres [10]–[18]. CENP-A overexpression in metazoans [19] and budding yeast [20] leads to its mislocalization. In budding yeast mislocalized Cse4 is very unstable [21]. Although budding yeast [22] and fission yeast [14], [23], [24] appear to be an exception, in several organisms CENP-A is loaded on the DNA outside of S phase, in anaphase of mitosis or the following G1 [25], [26], when it is proposed to replace histone H3.

Despite a significant progress in the field, the exact function of CENP-A at the centromere remains a mystery. CENP-A and H4 were reported to form a more compact and conformationally more rigid heterotetramer compared to the heterotetramer of histones H3 and H4 [6], [27]. However, the significance of the structural differences between H3 and CENP-A to their function is unknown. Even the question of the exact composition and localization of centromeric nucleosomes has not been resolved to date and remains the subject of controversy [28]. Besides an octamer composed of two molecules each of CENP-A, H2A, H2B and H4, a hexamer model in which Scm3 replaces H2A and H2B [11], [17] and a hemisome model which proposes a tetramer consisting of one copy each of Cse4, H4, H2A and H2B [29]–[32] were also proposed. Regional centromeres of higher eukaryotes can accommodate different versions of CENP-A-containing nucleosomes. While budding yeast with their point centromeres is an appealing model system to study the centromeric nucleosome, it is possible that the yeast centromeric nucleosome might also possess unique features.

Here we report the results of our analysis of the yeast centromeric nucleosome using a novel chromatin immunoprecipitation technique and discuss them in the context of the previously proposed models of the CENP-A containing nucleosome.

## Results

### High-resolution chromatin immunoprecipitation technique

The composition of the centromeric nucleosome was previously analyzed by means of chromatin immunoprecipitation (ChIP) [11], [12] in yeast. In a conventional ChIP approach proteins are chemically cross-linked to DNA, the chromatin is fragmented by sonication to about 500 bp size, and immunoprecipitated fragments are identified in PCR or microarray hybridization assays. This approach suffers certain drawbacks when applied to the centromere. The DNA fragment size is much larger than the region accommodated by a conventional nucleosome (146 bp), which limits the resolution. This problem can in principle be overcome by the treatment of chromatin with micrococcal nuclease, which specifically digests the internucleosomal linker DNA. However the size of kinetochore footprint is highly variable depending on the digest conditions [33], [34] and apparently poses an accessibility problem for antibodies since the efficiency of the co-immunoprecipitation of the CEN DNA with canonical histones is very low compared to pericentric regions [11], [12], [35]. In addition, PCR with a specific pair of primers or microarray hybridization detect larger DNA fragments without identifying them by size, which imposes further limits on resolution.

We developed new versions of ChIP to reveal the composition of the centromeric nucleosome in budding yeast. There are three main differences from conventional ChIP. First, we performed our experiments with and without the chromatin cross-linking. We reasoned that omitting cross-linking improves the accessibility of the centromeric nucleosome to antibodies and prevents potential artifacts due to the cross-linking of loosely associated proteins. However, because cross-linking prevents local re-arrangements due to nucleosomal sliding along the DNA, we also included cross-linked samples in our analysis. Second, we flanked CEN DNA by restriction sites and excised it by a specific endonuclease similar to earlier studies by [36]. Finally, analysis of the immunoprecipitated DNA was performed using methods that identify the isolated fragments by size, initially by a Southern blot with specific probes hybridizing to the excised CEN fragment. In experiments where qPCR with a specific pair of primers was used, the immunoprecipitated DNA was size-fractionated prior to PCR to preclude the detection of uncut DNA. The Biggins's laboratory recently employed a similar approach [37]. In this study, micrococcal-nuclease digested chromatin was immunoprecipitated with an anti-Cse4 antibody and analyzed by Southern blot. The results demonstrated a single Cse4 nucleosome positioned at the budding yeast centromere but did not address its composition further.

### Cse4 and H3 localize to a 214 bp CEN fragment

**Figure 1:**
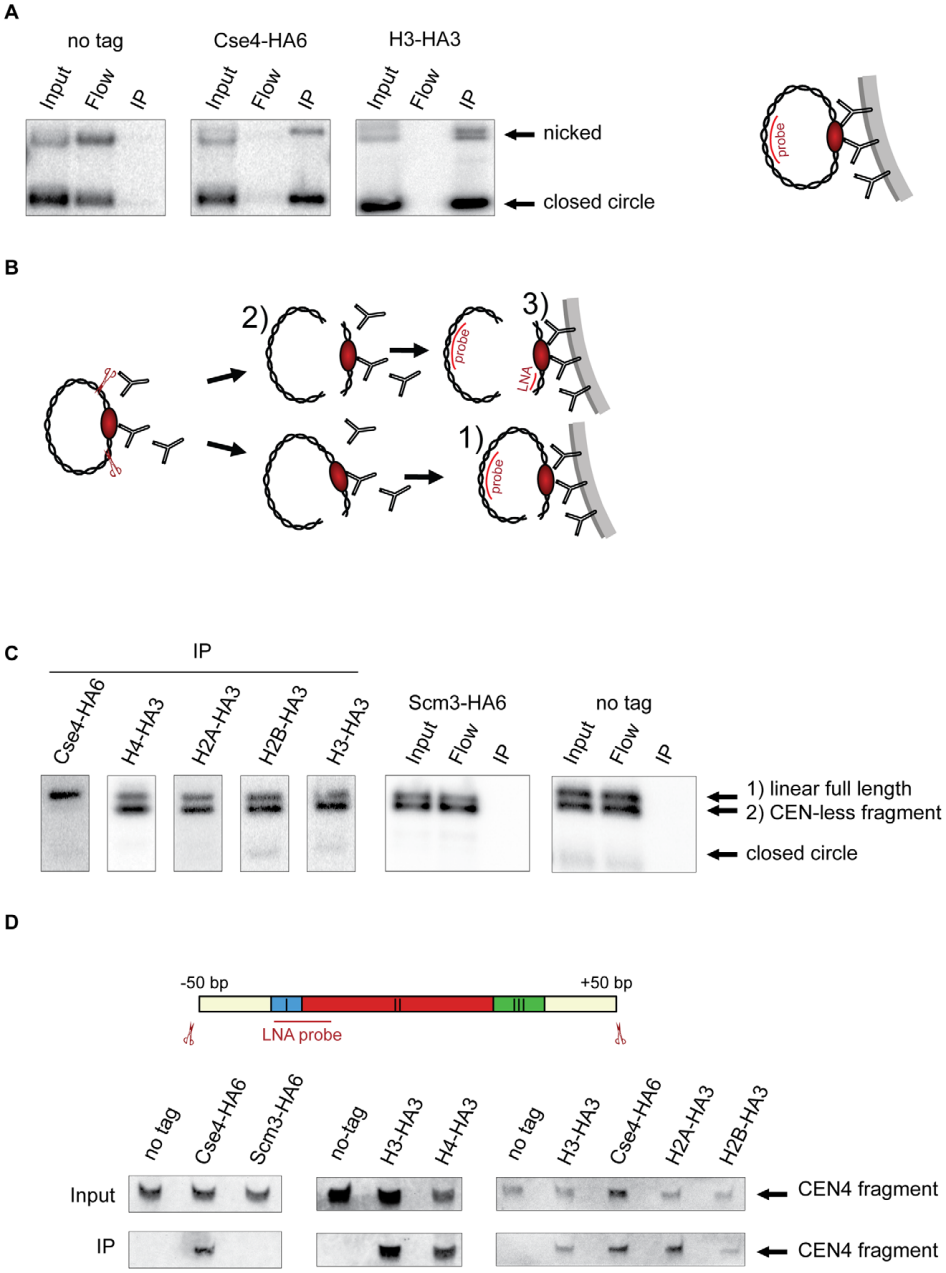
Composition of the centromeric nucleosome. A) The CEN-containing minichromosomes can be specifically co-immunoprecipitated with Cse4 and H3. Lysates from strains transformed with the minichromosomes 1021 (wt), 1498 (Cse4-HA6) and 1407 (H3-HA3) were incubated with anti-HA antibody and Dynabeads. DNA was eluted off the beads and separated on a 1% agarose gel. Southern blot was analyzed using a ^32^P labeled *TRP1* probe. The map of the minichromosome is shown in Figure S1. B) Experimental setup for the immunoprecipitation of minichromosomes digested with restriction enzyme. Chromatin is digested with BglII and incubated with anti-HA antibody recognizing tagged histones and protein A Dynabeads. Minichromosome digest with BglII produces three different fragments: a linearized full-length minichromosome (1), a CEN-less fragment (2) which can be detected with *TRP1* probe and a small CEN fragment (3) which can be detected with an LNA oligonucleotide. The red ellipse is depicting the centromeric nucleosome. C) Cse4 binding is restricted to minichromosomal CEN DNA. BglII-treated chromatin of strains carrying the minichromosome with BglII restriction sites 50 bp upstream and downstream of CEN boundaries was immunoprecipitated with anti-HA antibody. The strains were 1498 (Cse4-HA6), 1577 (H4-HA3), 1576 (H2A-HA3), 1587 (H2B-HA3), 1407 (H3-HA3), 1593 (Scm3-HA6), and 1021 (wt). DNA was analyzed as in (A) with ^32^P labeled *TRP1* probe. D) H3 is associated with the CEN DNA. Top: Scheme of the excised CEN fragment. Double-DIG labeled LNA probe for CDEI/II is indicated. Bottom: Immunoprecipitated DNA from experiments shown in (C) was separated on a 6% denaturing TBE polyacrylamide gel. Southern blot was analyzed using a double-DIG labeled LNA probe for CDEI/II. Western blots showing immunoprecipitation of the tagged proteins are shown in Figure S4A.

In our initial experiments we used a small minichromosome that contained the CEN region of chromosome IV (Figure S1A). We utilized strains with HA-tagged versions of H3 and Cse4 and found that the minichromosome can be specifically co-immunoprecipitated with an anti-HA antibody even in the absence of cross-linking (Figure 1A). This result demonstrates that the minichromosome assembles conventional nucleosomes as well as a centromeric nucleosome. Next, we tested whether it is possible to digest the minichromosome in yeast cell lysate and subsequently immunoprecipitate the fragments. We constructed minichromosomes with BglII sites at different positions with respect to CEN. The digest efficiency was highly variable depending on the position of the BglII site (Figure S1B). It was previously reported that the centromeric DNA is inaccessible for the nuclease digest [33], [34]. However, under our conditions it was possible to excise CEN DNA and even to cut it between CDEII and CDEIII in agreement with the previous results by [38], [39].

In subsequent ChIP experiments we used a minichromosome with BglII restriction sites 50 bp upstream and downstream of CEN4 boundaries flanking a 214 bp CEN fragment. The chromatin was digested with the endonuclease BglII and immunoprecipitated with an anti-HA antibody (Figure 1B). A probe hybridizing to the *TRP1* gene located on the minichromosome outside of CEN was used for the Southern blot. Due to an incomplete chromatin digest, a linearized full-length minichromosome and a CEN-less fragment could be detected. Only the full-length linearized minichromosome co-immunoprecipitated with Cse4-HA6 while both the full-length linearized minichromosome and the CEN-less fragment were recovered with HA-tagged histones H4, H2A, H2B and H3 (Figure 1C). Therefore, although the minichromosomes assemble conventional nucleosomes along their entire length, only CEN DNA is associated with Cse4, which is in agreement with [37]. Since it was proposed recently that the Scm3 histone chaperone might replace H2A/H2B dimers in the centromeric nucleosome [11], [17] we performed the minichromosome ChIP with the Scm3-HA6 strain. We could not co-immunoprecipitate the minichromosome with HA-tagged Scm3 under our conditions indicating that Scm3 is unlikely to be a part of the centromeric nucleosome (Figure 1C).

The observation that no CEN-less fragment was recovered in the Cse4-HA6 immunoprecipitation rules out lateral sliding of Cse4 nucleosome during the course of the immunoprecipitation as well as tethering of DNA fragments via protein-protein interactions, e.g., between centromeric and conventional nucleosomes in our assay. The efficiency of immunoprecipitation of the minichromosome fragments of approximately 1000 bp and longer was exceptionally high and close to 100%. When a 930 bp fragment from *ARS1* until position +50 downstream of CDEIII was excised, it could be depleted from yeast cell lysate with anti-HA antibodies recognizing Cse4-HA6 while virtually none of the remaining CEN-less fragment of the minichromosome could be detected on the beads (Figure S2). Considering the immediate proximity of the +50 cutting site to the centromere it is highly unlikely that there was a significant local rearrangement of nucleosomes and/or tethering of the CEN fragment to the rest of the minichromosome under our experimental conditions.

The detection of the small 214 bp CEN fragment was very inefficient using the ^32^P-labelled probe. Therefore we employed a digoxygenin (DIG)-labeled locked nucleic acid (LNA) oligonucleotide (Figure 1D) with improved hybridization properties [40]. Using the LNA probe it was possible to detect the 214 bp fragment released from 6 pg of the minichromosome which corresponds to about 0.1% efficiency of immunoprecipitation starting with 150 ml of yeast culture in the early log phase (Figure S3). We could detect the 214 bp CEN fragment in the immunoprecipitates with Cse4, H4, H2A and H2B. Surprisingly, we reproducibly observed an interaction of H3 with the 214 bp CEN fragment using this method (Figure 1D). This was in contrast with previous studies proposing that H3 is replaced by Cse4 at the centromere [2].

**Figure 2:**
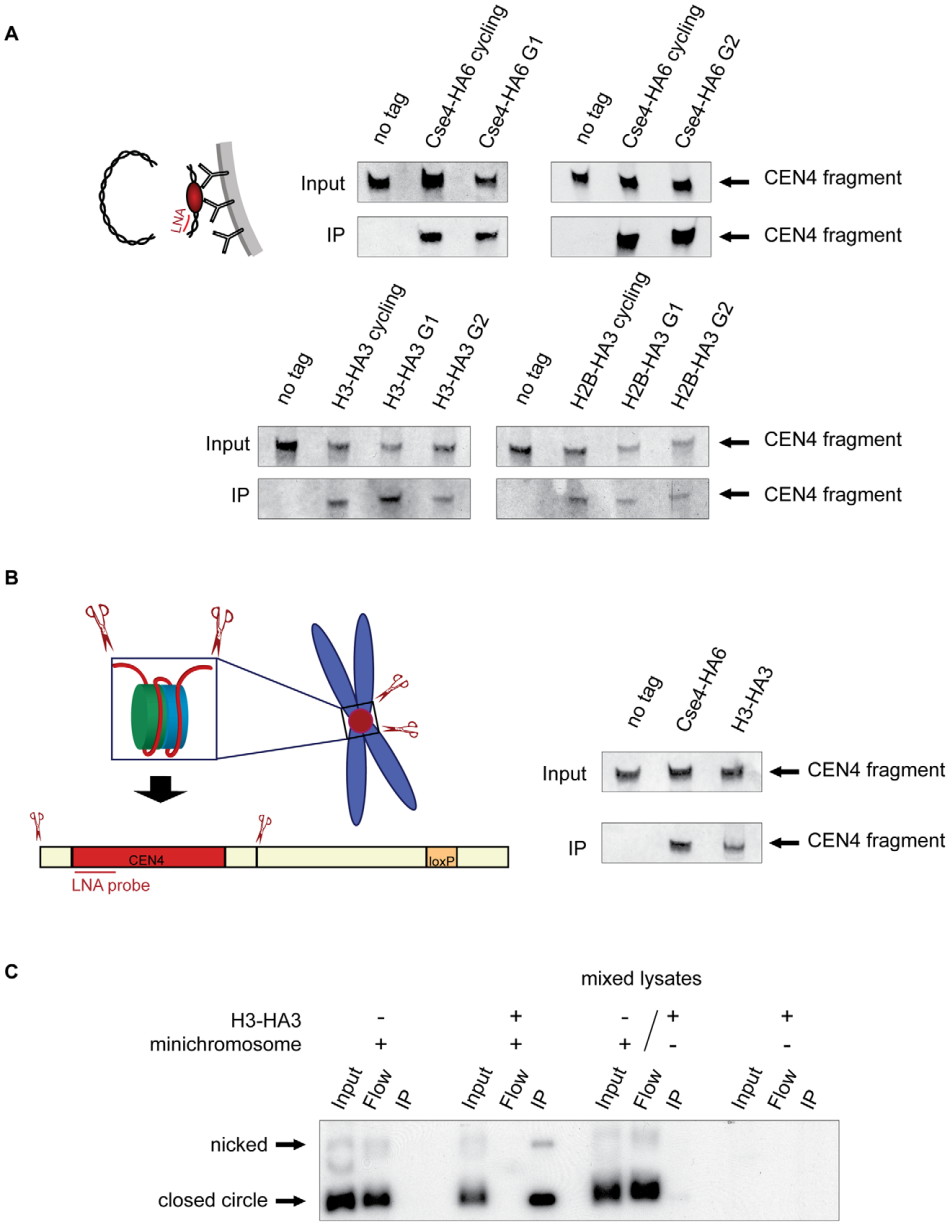
Histone H3 localizes to the centromeric DNA. A) H3 is associated with CEN DNA throughout the cell cycle. Strains carrying the minichromosomes with BglII restriction sites 50 bp upstream and downstream of CEN boundaries, 1498 (Cse4-HA6), 1407 (H3-HA3), and 1587 (H2B-HA3) were arrested in G1 with alpha factor and in G2 with nocodazole/benomyl. Chromatin was treated with BglII and immunoprecipitated with anti-HA antibody. DNA was eluted off the beads and resolved on a 6% denaturing TBE polyacrylamide gel. Southern blot was analyzed with a double-DIG labeled LNA probe for CDEI/II. The FACS profiles are shown in Figure S4B. B) H3 is associated with the CEN DNA on a native chromosome IV. BglII-treated chromatin of strains with BglII sites 50 bp upstream and downstream of CEN boundaries on chromosome IV 2059 (wt), 2043 (Cse4-HA3), and 2042 (H3-HA3) was immunoprecipitated with anti-HA antibody. DNA was eluted off the beads, separated on a 6% denaturing TBE polyacrylamide gel and analyzed with a double-DIG labeled LNA probe for CDEI/II. C) Minichromosome-bound histone H3 does not turn over during the immunoprecipitation procedure. Lysates of strains 1021 (wt, carrying the minichromosome), 1407 (H3-HA, carrying the minichromosome), 1407 (H3-HA3, without the minichromosome), and mixed lysate of 1021 (wt with minichromosome) and 1407 (H3-HA3, without the minichromosome) were incubated with anti-HA antibody and Dynabeads. DNA was eluted off the beads, separated on a 1% agarose gel and analyzed using a ^32^P labeled *TRP1* probe.

We next tested whether the interaction of H3 with CEN is dependent on the cell cycle stage as it is possible that Cse4 replaces H3 at a specific point in the cell cycle. The notion that the composition of the centromeric nucleosome might vary through the cell cycle was proposed earlier [17], [28]. Yeast cultures were arrested in G1-phase with alpha-factor and in G2-phase with nocodazole/benomyl (Figure S4B), and chromatin was digested with BglII to release the 214 bp CEN fragment prior to immunoprecipitation. Both H3 and Cse4, as well as H2B, were found to be associated with CEN in G1-phase and in G2-phase (Figure 2A).

Although nearly a 100% efficiency of co-immunoprecipitation of the minichromosomes with Cse4-HA6 (Figure 1A) indicated that it is unlikely to be the case, it is possible that a fraction of minichromosomes assemble a conventional nucleosome at the centromere and this would explain the association of H3 with CEN DNA in the above experiments. To address this possibility we adapted our ChIP approach to the native centromeres on the chromosomes and introduced BglII restriction sites 50 bp upstream and downstream of CEN on chromosome IV. The excised “native” 214 bp CEN4 fragment could be efficiently co-immunoprecipitated with H3-HA3 and Cse4-HA6 (Figure 2B). We conclude that both histones H3 and Cse4 localize to centromeric DNA in budding yeast.

In order to rule out the possibility that Cse4 is replaced by H3 during our immunoprecipitation procedure, we mixed yeast cell lysate of an H3-HA3 strain that does not carry minichromosomes with lysate of an untagged H3 strain carrying the minichromosomes. We could not observe any immunoprecipitation of the minichromosome with anti-HA antibody from those mixed lysates (Figure 2C). Thus there is little or no turnover of minichromosome-associated H3 in our cell lysates.

However, this experiment could not rule out local rearrangement of nucleosomes such as lateral sliding in the course of our experimental procedure, which included long incubations. Therefore we cross-linked proteins to DNA with formaldehyde prior to immunoprecipitation. Adding formaldehyde to the spheroplasts dramatically reduced the efficiency of centromeric DNA co-immunoprecipitation with either Cse4 or H3. This was partially due to the low yield of the minichromosome in the cleared lysate after centrifugation presumably because the minichromosomes were cross-linked to larger structures. However, when formaldehyde was added directly to yeast lysate the immunoprecipitation was not impeded. In order to minimize the potential rearrangement of nucleosomes after cell lysis, the duration of the restriction digest of the minichromosomes was limited to 5 minutes followed by formaldehyde addition and immunoprecipitation. We were able to efficiently co-immunoprecipitate the 214 bp CEN fragment with both Cse4 and H3 after cross-linking (Figure S5A). Therefore, it is unlikely that the detection of H3 at the CEN DNA is due to nucleosomal sliding during our experimental procedure.

**Figure 3:**
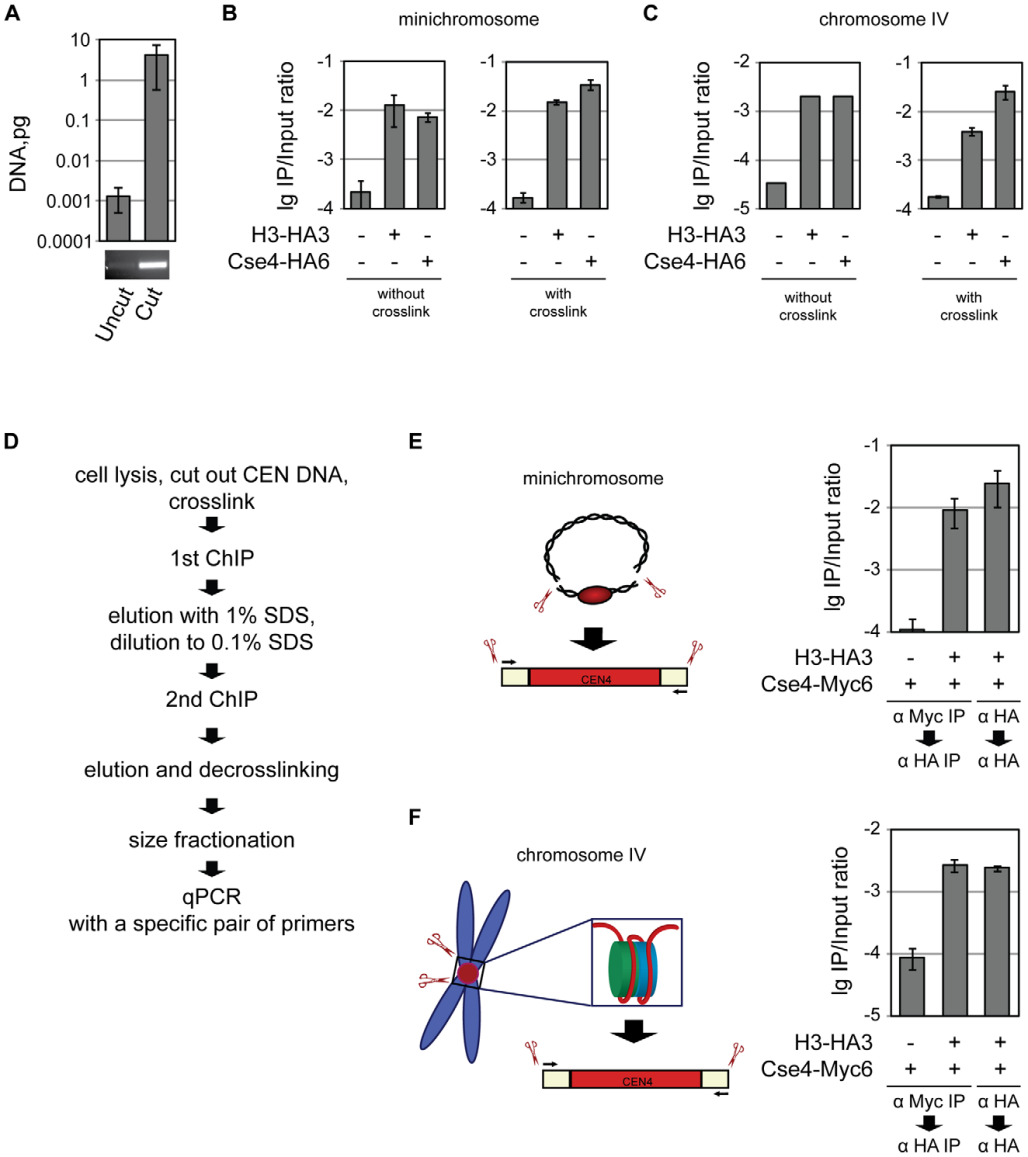
Co-occupancy of the centromeric DNA by histone H3 and Cse4. A) Only the 214 bp BglII CEN4 fragment and no full-length minichromosome is detected in the ChIP/qPCR assay. DNA isolated from untreated and BglII-treated lysates was size-fractionated on 2% agarose gel and analyzed by qPCR. A PCR product after 30 cycles of amplification in a conventional PCR reaction with the same primers that were used for qPCR is shown below. B) Minichromosomal CEN DNA can be co-immunoprecipitated with H3 and Cse4. BglII-treated chromatin of the strains 1021 (wt), 1407 (H3-HA3), and 1498 (Cse4-HA6) carrying the minichromosome was either not cross-linked or cross-linked with formaldehyde and immunoprecipitated with anti-HA antibody. The immunoprecipitated DNA was purified and size fractionated and subjected to qPCR analysis. C) CEN DNA of the native chromosome IV can be co-immunoprecipitatd with H3 and Cse4. BglII-treated chromatin of the strains 2059 (wt), 2042 (H3-HA3), and 2043 (Cse4-HA6) with CEN DNA of the native chromosome IV flanked with BglII was either not cross-linked or cross-linked with formaldehyde followed by immunoprecipitation as in (B). D) Flowchart of the sequential Cse4-H3 ChIP. E) Sequential ChIP of minichromosomal CEN DNA. BglII-treated chromatin of the strains 1923 (Cse4-Myc6) and 2300 (H3-HA3, Cse4-Myc6) carrying the minichromosome was cross-linked with formaldehyde and immunoprecipitated with anti-Myc or anti-HA antibody as indicated in the figure, the DNA was eluted off the beads and re-immunoprecipitated with anti-HA antibody. The immunoprecipitated DNA was purified, size fractionated on a 2% agarose gel and subjected to qPCR analysis. F) The same as in (E) but performed with the native CEN DNA. The strains, 2562 (Cse4-Myc6), and 2561 (H3-HA3, Cse4-Myc6) had CEN DNA of the native chromosome IV flanked with BglII. The bar graphs represent the average values from several independent experiments with SDs.

A qPCR-based approach was employed to compare the efficiencies of co-immunoprecipitation of the CEN DNA with H3-HA3 and Cse4-HA6. After excision of the 214 bp CEN fragment CEN DNA was co-immunoprecipitated with Cse4-HA or H3-HA using anti-HA antibodies, eluted off the beads using SDS, size-fractionated via agarose gel-electrophoresis to separate it from full-length minichromosome and quantified using a quantitative PCR reaction. Using this procedure, we ensured that the 214 bp CEN fragment was exclusively detected since no PCR product was obtained when the restriction digest step was omitted (Figure 3A). We did not observe any significant differences in ChIP efficiencies with H3 and Cse4 when the same anti-HA antibody was used. Similar IP/input ratios were observed with and without crosslink (Figure 3B) with the CEN DNA located on a minichromosome and on the native chromosome IV flanked by restriction sites (Figure 3C). Thus we have no indication that only some centromeres are associated with H3.

### Co-occupancy of the centromeric DNA by histone H3 and Cse4

The association of H3 and Cse4 with yeast centromeres can be mutually exclusive, i.e., a fraction of the centromeres are occupied by the Cse4 nucleosome while a different fraction assembles a conventional nucleosome containing H3. Alternatively, H3 and Cse4 are co-occupying the centromeric DNA at the same time. In order to distinguish between these two possibilities we performed a sequential ChIP experiment. After excision of the 214 bp CEN fragment and formaldehyde cross-linking CEN DNA was co-immunoprecipitated with Cse4-Myc using anti-Myc antibodies covalently coupled to the beads (Figure S5B and S5C), eluted off the beads using SDS, and re-immunoprecipitated with anti-HA antibodies recognizing H3-HA. The CEN DNA fragment eluted off the beads was decross-linked, size-fractionated via agarose gel-electrophoresis to separate it from uncut DNA, and quantified using a quantitative PCR reaction (Figure 3D–3F). The efficiency of the second immunoprecipitation step in this experiment was approximately 100 fold higher than the “mock” immunoprecipitation from a strain in which only Cse4 was tagged and was comparable to that of H3-HA re-immunoprecipitation in the experiment where both the first and the second steps were performed with anti-HA antibodies. Similar results were obtained when CEN DNA was excised from the minichromosome (Figure 3E) or native chromosome (Figure 3F). We conclude that H3 and Cse4 co-exist at least at some centromeres. Unfortunately, we could not perform the reverse experiment, i.e., to immunoprecipitate the CEN DNA via HA-tagged histone H3 and then re-precipitate via Myc-tagged Cse4, since we could not re-precipitate CEN DNA from Cse4-Myc strain with anti-Myc antibody in 0.1% SDS. Switching the tags was also unsuccessful since the H3-Myc6 strain was not viable.

### Is the centromeric nucleosome a heterotypic octamer?

Because the length of our excised centromeric fragment (214 bp) is much shorter than would be necessary to accommodate two conventional nucleosomes (292 bp assuming no linker DNA in-between) or a conventional nucleosome and a Cse4 nucleosome (268 bp if the Cse4 nucleosome organizes only 121 bp of DNA [41]), it is plausible that the centromeric nucleosome is a heterotypic octamer with one molecule of H3 and one molecule of Cse4. If the structure of this hypothetical heterotypic nucleosome is similar to the structure of the conventional nucleosome and the CENP-A containing nucleosome [41], [42], histones H3 and Cse4 are expected to form a four-helix bundle with parts of their α2 and α3 helices. In vertebrates and many other organisms the α2 helix of H3 contains a cysteine residue, C110. These cysteine residues from two histones H3 within the same nucleosome are within 6.2 Å from each other [42] and were reported to form a disulfide bond under oxidizing conditions in vitro [43]. In human CENP-A the corresponding residue is a leucine, L112, although CENP-A proteins from some other mammals, such as platypus, as well as birds and amphibians have a cysteine in this position. In the recently reported crystal structures of human CENP-A nucleosome the two leucines 112 are 4.8–5.7 Å apart [27], [41], which should allow cross-linking if they are mutated to cysteines. (Figure S6A). In order to test whether a cross-link between two Cse4 molecules or between Cse4 and H3 is at all possible we co-expressed the histone fold domain of Cse4-Cys and the full-length H3-Cys in bacteria. We could observe the formation of spontaneous covalently cross-linked H3 homodimers, Cse4 homodimers and some H3/Cse4 heterodimers. The dimers were detected after denaturing SDS-electrophoresis and could be resolved by β-mercaptoethanol treatment indicating that they indeed resulted from the formation of the disulfide bond between the cysteine residues (Figure S6B).

We reasoned that disulfide bond formation between the two α2 helix cysteines would only be possible if the two histones form a four helix bundle and the ability to cross-link Cse4 and H3 would be a test of a heterotypic octamer model. Since in budding yeast neither H3 nor Cse4 contain cysteine residues, we mutated the corresponding alanine 111 and leucine 204 to cysteines. We were able to cross-link homodimers of H3-Cys in crude lysates and on isolated chromatin in the presence of 5,5′-dithiobis-(2-nitrobenzoic acid) (DTNB, Ellman's reagent), which has been reported to facilitate intermolecular disulfide bond formation between H3 histones in chicken nucleosomes [44] (Figure S7A). We could also cross-link H3-Cys histones using cysteine-specific cross-linkers, bBBr and BMOE. However, we did not observe a reproducible cross-link either between two Cse4-Cys molecules or between Cse4-Cys and H3-Cys (Figure S7B) in crude yeast lysate or isolated chromatin.

Thus we currently have no direct evidence for the presence of the heterotypic octamer at budding yeast centromeres. It is possible that the heterotypic nucleosome has a very unusual structure compared to the conventional H3-H3 nucleosome [42] or the human CENP-A-CENP-A octamer that were recently reported [27], [41] and that this structure does not allow for the cysteine cross-link. It remains to be confirmed whether the cysteines can be cross-linked in the context of the fully assembled octamers.

### Cse4 and histone H3 do not occupy separate sub-regions within the centromeric DNA

An alternative to the octamer is the hemisome model, which proposes a tetramer consisting of Cse4, H4, H2A and H2B histones [30], [31]. Our refinement of this model will imply that in budding yeast in the immediate vicinity of the Cse4 hemisome there is either a conventional nucleosome or, possibly, an H3-containing hemisome. According to the recently reported structure, the human CENP-A-containing octamer assembled in vitro organizes 121 bp of DNA [41] while a conventional nucleosome wraps 147 bp of DNA. Thus, a Cse4 hemisome and a conventional nucleosome without any linker in-between would require approximately 207 bp which would fit with the size of our excised centromeric fragment of 214 bp. An important and testable prediction of this model is that Cse4 and histone H3 are incorporated into distinct structures, which can be potentially mapped to different stretches of DNA.

**Figure 4:**
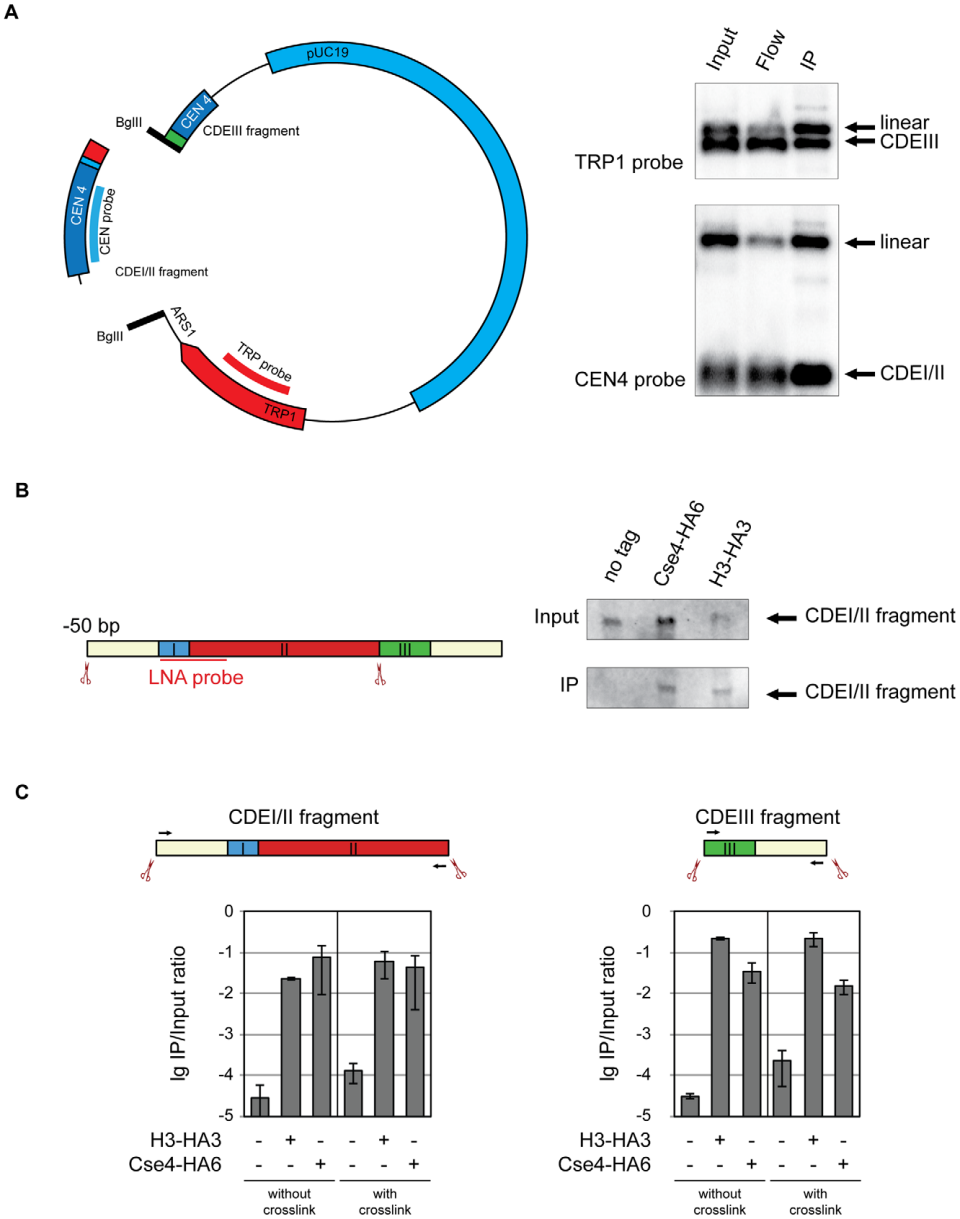
Cse4 association with CDEI/II and CDEIII. A) Cse4 nucleosome straddles the boundary between CDEII and CDEIII. Left: Map of the minichromosome utilized in the experiment. The construct contains 850 bp of pericentromeric sequence of chromosome IV, *TRP1* marker, *ARS1* and pUC19 sequence and has a size of 4.5 kb. There are two BglII sites: between CDEII and CDEIII in the CEN and in the *ARS1*. Right: BglII-treated chromatin of a strain 1498 (Cse4-HA6) carrying the minichromosome was immunoprecipitated with anti-HA antibody. DNA was eluted off the beads and separated on a 1% agarose gel. Southern blot was analyzed with a ^32^P labeled probe for the pericentric CEN4 sequence (to detect the CDEI/II containing fragment) and a ^32^P labeled probe for the *TRP1* gene (to detect the CDEIII containing fragment). B) Both Cse4 and H3 are associated with the CDEI/II fragment. Left: Scheme of CDEI/II fragment excised from the minichromosome. Double-DIG labeled LNA probe for CDEI/II is indicated. Right: BglII-treated chromatin of strain 1498 (Cse4-HA6) and 1407 (H3-HA3) carrying the minichromosome with BglII sites between CDEII and CDEIII and 50 bp upstream of CDEI was cross-linked with formaldehyde and immunoprecipitated with anti-HA antibody. DNA was eluted off the beads and resolved on a 6% denaturing TBE polyacrylamide gel. Southern blot was analyzed with a double-DIG labeled LNA probe for CDEI/II. C) Both the CDEI/II and the CDEIII fragments can be co-immunoprecipitated with Cse4 and H3. Strains 1021 (wt), 1407 (H3-HA3), and 1498 (Cse4-HA6) carried the minichromosome where either the CDEI/II (left) or the CDEIII fragment (right) was flanked with BglII sites. BglII-treated chromatin was either not cross-linked or cross-linked with formaldehyde and immunoprecipitated with anti-HA antibody. The immunoprecipitated DNA was purified, size fractionated, and subjected to qPCR analysis. Bar graphs represent the average values from several independent experiments with SDs.

The budding yeast centromere is defined by a 125 bp sequence [45] consisting of three elements. CDEI is a non-essential 8 bp palindrome, CDEII is 78–86 bp long and is composed of 87–98% A/T, and CDEIII is a highly conserved 25 bp sequence which binds the CBF3 protein complex [46]. We conducted a series of experiments in which we tested whether Cse4 and histone H3 associate with distinct elements within CEN DNA. It was reported earlier that *CSE4* genetically interacts with CDEI and CDEII but not with CDEIII [47] suggesting that the Cse4-containing nucleosome is localized upstream of the CDEII/CDEIII boundary. Since we were able to cut the minichromosome between CDEII and CDEIII we hoped to gain further insights in the exact localization of Cse4 with regard to CEN by using our ChIP approach. We created a minichromosome with a restriction site between CDEII and CDEIII and a restriction site outside of the CEN DNA, in *ARS1*. Using our ChIP approach we were able to co-immunoprecipitate Cse4-HA6 with both the CDEI/CDEII and the CDEIII-containing fragments (Figure 4A) suggesting that the centromeric nucleosome straddles the boundary between CDEII and CDEIII. However, an interaction with the CDEIII fragment appeared less efficient, indicating that the Cse4-containing nucleosome interacts mostly with the CDEI/CDEII region of the CEN DNA. An important corollary from this observation is that in our assay the Cse4-containing nucleosome (or hemisome) is not displaced from the CEN DNA to the edge of the 214 bp fragment.

To gain further insight into spatial distribution of H3 and Cse4-containing nucleosomes on CEN DNA we next excised a 139 bp fragment from position −50 upstream of CDEI until the CDEII/CDEIII boundary. When cross-linked, this fragment could be co-immunoprecipitated with both H3 and Cse4 (Figure 4B). This result demonstrates that H3 is present at the CDEI/II region of the centromere and/or at the preceding 50 bp of the non-centromeric DNA. Since the detection of a fragment containing CDEIII and 50 bp of DNA downstream of the CEN DNA with the LNA probe was not possible, we followed the association of histone H3 and Cse4 with CDEI/II and CDEIII elements using qPCR. Both the fragment containing CDEI/II region with upstream 50 bp and the fragment containing CDEIII region with the downstream 50 bp could be co-immunoprecipitated with HA-tagged Cse4 and histone H3 with and without crosslinking (Figure 4C). Therefore histone H3 and Cse4 appeared to be inseparable when associated with the CEN DNA implying that they are likely to be a part of one and the same structure. We would like to note that since Cse4 is capable of tethering CDEII and CDEIII fragments together (Figure 4A), the co-immunoprecipitation of the small CDEI/II and CDEIII fragments with H3 might be due to the small CDE-containing fragments maintaining the association with the large CDE-less fragment of the minichromosome throughout co-immunoprecipitation. No such tethering was observed when the complete 214 bp CEN DNA containing fragment was excised from the minichromosome (Figure 1C and Figure S2).

## Discussion

Three models of the centromeric nucleosome are proposed in the literature. In the first model the centromeric nucleosome is an octamer, where Cse4/CENP-A replaces histone H3. While octameric nucleosomes with two copies of budding yeast Cse4 [48], [49] or human CENP-A [41] were assembled in vitro, whether only one or both copies of H3 are replaced in vivo is not known. There is evidence from different organisms for and against either of these possibilities. In HeLa cells CENP-A released from chromatin by micrococcal nuclease digestion is still associated with histone H3 even after 2M NaCl treatment resulting in dissociation of H2A and H2B, implying heterotypic tetramers with two histones H4, one H3 and one CENP-A [4]. In contrast, in *Drosophila* S2 and Kc cells when chromatin is digested with micrococcal nuclease and CENP-A/CID is immunoprecipitated, no H3 co-purifies with CENP-A [1]. It was recently reported that *Drosophila* CENP-A/CID forms homodimers in vivo, which are unexpectedly very salt-sensitive but could be crosslinked via cysteines in the four-helix bundle after a prolonged incubation [50]. The authors did not exclude the formation of H3-CENP-A/CID heterodimers in addition to CENP-A/CID homodimers and it remains possible that different forms of CENP-A/CID nucleosomes are simultaneously present at the regional centromeres of *Drosophila* and possibly other higher eukaryotes.

**Figure 5:**
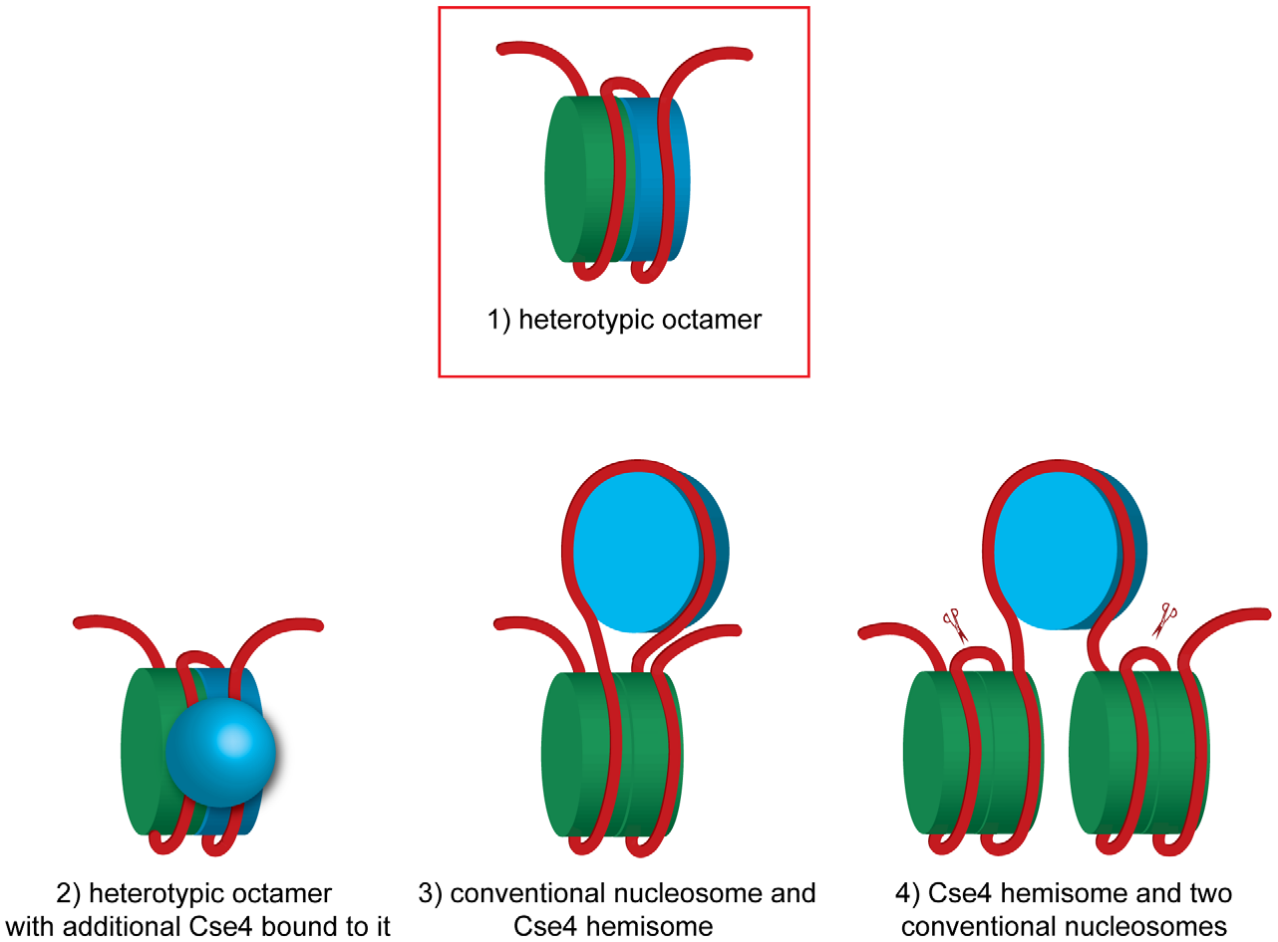
Models of how H3 and Cse4 can co-occupy the centromeric DNA. A heterotetramer of H3, H2A, H2B and H4 is colored in green and a heterotetramer containing Cse4 instead of H3 is blue.1) A heterotypic octamer containing both Cse4 and H3. 2) A heterotypic octamer with additional Cse4 bound to it. 3) A Cse4 hemisome incorporated in the loop of a conventional nucleosome. A DNA fragment of 207 bp is sufficient to accommodate this arrangement (without spacer DNA). 4) Two conventional nucleosomes flanking a Cse4 hemisome. The scissors indicate the BglII sites flanking the 214 bp fragment excised in our experiment. In case of model 4 this fragment would be tethered to non-centromeric DNA. The tethering was not observed in our experiments (Figure 1C). See text for discussion and additional details.

In this study we demonstrate that a budding yeast centromeric DNA fragment of only 214 bp is associated in vivo with both H3 and Cse4. We can exclude a homotypic octamer with two copies of Cse4. Our experiments suggest a very intimate spatial association between the conventional histone H3 and centromeric Cse4. This association cannot be explained if the Cse4-containing centromeric nucleosome is separated from the neighboring conventional H3 nucleosomes by spacer DNA as was proposed recently [51] but rather suggests that H3 and Cse4 co-occupy the CEN DNA fragment of only 214 bp in length. We favor the Cse4-H3 heterotypic octamer model (Figure 5, model 1). This octamer appears to be resistant to cysteine cross-linking, which might be due to the reduced stability of the four-helix bundle similar to the *Drosophila* CENP-A/CID [50].

The hexamer model postulates that in budding yeast the non-histone protein Scm3 replaces H2A and H2B and the nucleosome is composed of two copies each of Scm3, CENP-A and H4 [11], [17]. Although it was initially proposed that the Scm3 dimer constitutes an integral part of the centromeric hexasome [11], the recent structures of budding yeast Scm3 associated with Cse4/H4 [16], [18] and human HJURP in complex with CENP-A/H4 [52], [53] revealed that binding of DNA as well as the (Cse4/H4)_2_ heterotetramer formation are incompatible with Scm3 binding. In the experiments in vitro it was demonstrated that Scm3 association with the reconstituted (Cse4/H4)_2_ nucleosome-like particles depends on a DNA binding domain within Scm3 [17]. Our results are compatible with the view that Scm3 does not form a part of the centromeric nucleosome. Under our experimental conditions we were able to co-immunoprecipitate minichromosomes with Cse4, H4, H2A, H2B and H3 but not with Scm3, which most likely dissociated from the centromere in yeast lysate.

Finally, the hemisome model proposes a tetramer consisting of Cse4, H4, H2A and H2B histones [30]–[32]. According to this model, the Cse4 hemisome is positioned mostly at CDEII [20] and is expected to occupy approximately 60 bp of DNA [41]. This scenario leaves approximately 77 bp on each side of our 214 bp fragment available to accommodate the H3-containing nucleosome(s). We can speculate that a hemisome with Cse4 might, for example, be incorporated into a DNA loop between the two halves of an H3-containing octamer (Figure 5, model 3). This model might explain the tripartite organization of the budding yeast centromere that was observed in the micrococcal nuclease protection pattern [20]. Although it is technically possible that 77 bp upstream and downstream of the hypothetical centromeric hemisome are wrapped around ½ of the flanking conventional nucleosomes (Figure 5, model 4), this model will result in tethering of the excised 214 bp fragment to the rest of the minichromosome which we did not observe (Figure 1C and Figure S2) and therefore can be excluded.

More exotic models can be also considered. Two recent studies compared Cse4-GFP fluorescence in vivo to independent standards and found 3.5–6.0 [54] or even 7.6 [55] Cse4-GFP molecules per budding yeast centromere in anaphase. Even more surprisingly, in prolonged G1 arrest Cse4-GFP fluorescence was reduced more than two-fold [55]. These observations are inconsistent with the notion of a single Cse4 nucleosome at the budding yeast centromere [37]. It was proposed that the budding yeast centromere is in fact a regional centromere with additional Cse4s associated with the flanking DNA similar to the much larger centromeres of higher eukaryotes [54]. However, we could not observe any Cse4 associated with the non-centromeric part of the 2.4 kb minichromosome, which is expected to assemble 10 conventional nucleosomes. Therefore no additional Cse4 nucleosomes assemble, at least at these relatively short flanking sequences. Our results are consistent with those of [20], [56] who did not detect additional Cse4 nucleosomes in centromere-flanking regions by high-resolution mapping of yeast genome. The additional Cse4 molecules at the centromere could result from Cse4 mis-incorporation which is observed in strains overexpressing Cse4 [20] and could potentially be caused by GFP-tagging. Alternatively, additional Cse4 molecules may not be incorporated into the centromeric nucleosome but are rather associated with it via protein-protein and/or protein-DNA interactions (Figure 5, model 2). In this scenario the centromeric nucleosome can be a Cse4-H3 heterotypic octamer to which more Cse4 molecules are bound. Intriguingly, when (Cse4/H4)_2_ heterotetramers were reconstituted in the presence of Scm3 into nucleosome-like particles on a 207 bp-long high affinity nucleosome positioning DNA sequence in vitro, high molecular weight complexes possibly representing additional Cse4/H4 in loose association with the Cse4/H4/DNA complex were detected [49]. Similar complexes were reported to be assembled in vitro on a 148 bp CEN3 DNA [17].

It is more than a decade now since it was proposed that H3 is replaced by the histone variant Cse4 [2]. Our results appear to contradict this well-established dogma. If Cse4 and H3 indeed co-localize to the centromeric DNA why wasn't it noticed before? We can offer the following explanation. We have noticed that in most publications reporting ChIP experiments at the budding yeast centromere, the absolute efficiency of ChIP of the CEN DNA with H3 and Cse4 is very similar and typically in the range of 1% [11], [35]. The claim that only Cse4 is associated with the CEN DNA is then based on an observation that non-centromeric DNA is co-immunoprecipitated with H3 at about 5 to 10-fold higher rate than CEN DNA while almost no non-CEN DNA is found associated with Cse4 (Figure S8). We suggest that if CEN DNA were generally difficult to immunoprecipitate, for example due to cross-linking of the large number of kinetochore proteins during the in vivo cross-linking, this would explain the reduced efficiency of H3 ChIP at the centromere compared to the chromosomal arms.

Our results appear to contradict those of [35]. This group could co-immunoprecipitate differentially tagged versions of Cse4 from budding yeast but did not observe co-immunoprecipitation of tagged Cse4 and H3. However, one of the tagged Cse4s was expressed from a plasmid and Cse4 overexpression was reported to result in its ectopic incorporation genome-wide into octameric nucleosomes that were not observed in the wild type strain [20]. It remains possible that even in budding yeast there is a degree of heterogeneity in the composition of the centromeric nucleosomes among different chromosomes and that either a homotypic Cse4/Cse4 octamer or a heterotypic Cse4/H3 octamer can provide the essential function.

At this time we can only speculate at the function of H3 at the budding yeast point centromere. It is possible that the presence of two different nucleosomes (or hemisomes), one with Cse4 and one with H3 provides structural asymmetry which might form the basis for two separate surfaces, one facing the sister centromere and another providing the attachment site for the spindle microtubule.

## Materials and Methods

### Plasmids and strains

Generation of the minichromosome containing a 850 bp long sequence from chromosome IV encompassing CEN4 was described earlier [57], [58]. A version without Tet operators was used to introduce BglII restriction sites using QuikChange Site-Directed Mutagenesis Kit (Stratagene). A SalI digest and religation was used to remove the pUC19 sequence from the final construct prior to transformation into yeast.

To introduce BglII restriction sites flanking the CEN DNA into the native chromosome IV, the region of CEN4 +/− 200 bp was cloned into the PvuII site of pOM10 (courtesy of Anne Spang) and BglII sites were introduced by mutagenesis. A yeast strain was transformed with a PCR product containing CEN4 DNA with BglII sites, marker, and a CEN flanking sequence. The BglII flanked CEN4 DNA was recombined into the endogenous locus and the marker cassette was removed with Cre recombinase [59] leaving 85 bp of the pOM10/loxP sequence 200 bp downstream of CDEIII (Figure 2B). The whole CEN4 region was sequenced.

Cse4 was tagged with HA6, Myc6 or Myc3 at an internal XbaI site as described in [2]. All other histones were tagged at the C-terminus and the second gene was either left untagged (H4) or deleted (H2A, H2B, H3). The strains are described in Table S1.

### Chromatin immunoprecipitation

Yeast strains transformed with the minichromosome were grown overnight in synthetic medium without tryptophan at 30°C, were inoculated into fresh medium to a final OD_600_ of 0.2, and grown until the OD_600_ reached 1.6. For G1 arrest, yeast culture was grown from an OD_600_ of 0.05 until an OD_600_ of 0.2 and then arrested with 2 µg/ml alpha factor for 1 hour. After 1 hour, additional 1.5 µg/ml alpha factor was added followed by an additional hour of incubation. For G2/M arrest, 15 µg/ml nocodazole and 10 µg/ml benomyl were added to a yeast culture at an OD_600_ of 0.65 in YEPD medium, and cells were incubated for 1.5 hours.

Spheroplasting was carried out with lyticase (Sigma, L2524) as described in [60]. Spheroplasts were lysed for 30 min on ice in 2.5 ml of lysis buffer (25 mM HEPES/KOH [pH 8.0], 50 mM KCl, 10 mM MgSO_4_, 10 mM Na citrate, 25 mM Na sulfite, 0.25% TritonX-100, 1 mM PMSF, 3 mM DTT, 1× complete EDTA-free protease inhibitors (Roche) and 100 µg/ml RNase A). The lysate was cleared by centrifugation at 10,000 rpm for 5 min in an Eppendorf microcentrifuge. For DNA cleavage, lysate was incubated with 1 unit/µl of BglII (NEB) for 2 hours with rotation at 4°C before adding NaCl to a final concentration of 300 mM to stop the digest. For strains with BglII sites on chromosome IV the crude lysate was incubated with BglII and cleared after 2 hours of digestion. Pre-cleared lysate (2 ml) was incubated with 25 µg of anti-HA (12CA5) antibody and 0.5 ml suspension of protein A Dynabeads (Invitrogen) overnight. Beads were washed 3 times with 1.5 ml of the lysis buffer with 300 mM NaCl. Isolated DNA was eluted off the beads two times with 250 µl of 50 mM Tris [pH 8.0], 10 mM EDTA and 1% SDS at 65°C. For cross-linked chromatin the DNA digest with BglII was performed for 5 min at 37°C, the digest was stopped by adding 300 mM NaCl and chromatin was cross-linked by adding 0.1% formaldehyde for 30 min and 125 mM glycine for 15 min on ice. The cross-linked lysate was incubated with protein A Dynabeads covalently coupled to either anti-HA (12CA5) or anti-Myc (9E11) antibody with DMP (dimethyl pimelimidate) according to the manufacturer's guidelines. For the sequential immunoprecipitation the chromatin was eluted off the beads as described above, diluted to 0.1% SDS with lysis buffer with 300 mM NaCl and immunoprecipitated with protein A Dynabeads covalently coupled to anti-HA (12CA5). The DNA was eluted off the beads as above. All the samples were adjusted to 1% SDS final concentration, extracted twice with phenol/chloroform/isoamyl alcohol (25∶24∶1), ethanol precipitated in the presence of 20 µg glycogen (Roche) and samples were dissolved in 20–40 µl TE. For the Southern blots detected with a ^32^P-labelled probe specific for *TRP1* or CEN4, samples were separated on a 1% agarose gel with ethidium bromide and a capillary transfer to Hybond-N+ (GE) was carried out under neutral conditions. Blots were scanned on Personal Molecular Imager (Bio-Rad) and bands quantified with QuantityOne 4.6.7. For Southern blots detected with double-DIG labeled LNA probe (AAAGTTGATTATAAGCATGTGAC, Exiqon) samples were separated on a denaturing 6% TBE polyacrylamide gel followed by an electrophoretic transfer to Hybond-N+ at 80 V for 1 hr in 1× TBE in the Trans-Blot System (Biorad). Hybridization with DIG labeled LNA probe was performed according to instructions of DIG High Prime DNA Labeling and Detection Starter Kit II (Roche). For qPCR the samples were size fractionated on a 2% agarose gel (Certified Low Range Ultra Agarose, Bio-Rad), gel excised to separate from uncut and linear minichromosome and subjected to qPCR with the primers AGTAACTTTTGCCTAAATCAC and TAGGTAGTGCTTTTTTTCCA for the 214 bp CEN4, TAGTAACTTTTGCCTAAATC and TAATAAATAAATTATTTCATTTATGTTT for the 139 bp CDEI/II fragment, and TGTTTATGATTACCGAAACA and TTAGGTAGTGCTTTTTTTCC for the 77 bp CDEIII fragment, qPCR analysis was performed using LightCycler 480 SYBR Green I Master (Roche) according to the manufacturer's manual.

### Ex vivo cross-linking of histones on chromatin

Spheroplasting was carried out using the same procedure as for ChIP. Spheroplasts were washed in 1 M sorbitol and lysed in cold reaction buffer (25 mM Sodium Phosphate [pH 7.0], 100 mM KCl, 2.5 mM MgCl_2_, 0.25% TritonX-100) for 15 min on ice. Chromatin was pelleted using a low-speed centrifugation (4,000 rpm, 1 min) and the supernatant was discarded. The chromatin pellet was then resuspended in the reaction buffer with varying concentrations of the cross-linker. DTNB (5,5′-dithiobis-(2-nitrobenzoic acid), Sigma) was prepared as a 50 mM stock in DMSO and diluted into the reaction mixture as appropriate. Cross-linking was allowed to proceed for 1 hour on ice. The chromatin was pelleted by centrifugation and resuspended in SDS-PAGE loading dye without DTT or β-mercaptoethanol.

### Protein expression in *E. coli*

Codon optimized sequences of yeast histone H3-Cys, N-terminally tagged with Avitag (Avidity), and the histone fold domain of Cse4-Cys (D150-end), N-terminally tagged with 6xHis, were cloned either together into pRSFDuet1 (Novagen) or separately, Cse4 in pETDuet1 and H3 in pRSFDuet1, transformed and expressed in BL21 (DE3) according to the manufacturer's instructions. Aliquots of bacterial culture were harvested and resuspended in SDS-PAGE loading buffer with and without β-mercaptoethanol. Samples were separated on a 15% SDS-PAGE and Western blots were analyzed with Streptavidin-HRP (Pierce) for H3-Cys and with anti-Penta-His antibody (Qiagen) for Cse4-Cys.

## Supporting Information

Figure S1 Accessibility of restriction endonuclease sites in the centromeric region of the minichromosome. A) Map of the minichromosome. The construct contains 850 bp of pericentromeric sequence of chromosome IV, *TRP1* marker and *ARS1*. B) Top: Scheme of CEN4 with CDEI, CDEII and CDEIII indicated. The scissors indicate BglII sites in the different constructs. Bottom: The efficiency of a minichromosome digest at the indicated sites. DNA was isolated from BglII-treated lysates of strains carrying different minichromosomes, resolved on a 1% agarose gel and analyzed with a ^32^P labeled *TRP1* probe.
(TIF)

Figure S2 Cse4 nucleosome remains restricted to the CEN DNA in the course of immunoprecipitation procedure. Top: Map of the minichromosome utilized in the experiment. The construct contains 850 bp of pericentromeric sequence of chromosome IV, *TRP1* marker and *ARS1*. BglII restriction sites are located 50 bp downstream of CDEIII and in *ARS1* and are indicated with scissors. Bottom: BglII-treated chromatin of a strain 1498 (Cse4-HA6) carrying the minichromomosome was immunoprecipitated with anti-HA antibody without cross-linking. A long version of the procedure with 2 hours restriction digest was used. The DNA was eluted off the beads, purified via phenol/chloroform extraction and ethanol precipitation and separated on a 1% agarose gel. Southern blot was analyzed with a *TRP1* probe to detect CEN-less fragment and a CEN4 probe hybridizing to the pericentromeric sequence to detect a fragment of the minichromosome containing CEN4.
(TIF)

Figure S3 Sensitivity of the Southern blot detection with double DIG-labeled LNA probe for CDEI/II. DNA purified from BglII-treated lysate of a strain 1021 carrying the minichromosome with BglII restriction sites 50 bp upstream and downstream of CEN4 and known quantities of the minichromosome purified from bacteria (miniprep) and digested with BglII were resolved on a 6% denaturing TBE polyacrylamide gel and analyzed by Southern blot with the LNA probe for CDEI/II.
(TIF)

Figure S4 (A) Anti-HA Western blots of samples from ChIP experiments. Input, unbound fraction and eluted beads were separated on SDS-PAGE. (B) FACS analysis of the arrested yeast cultures in the experiment in Figure 2A.
(TIF)

Figure S5 ChIP of minichromosomal and native CEN DNA fragment after formaldehyde cross-link. A) BglII-treated chromatin of the strains 1021 (wt), 1407 (H3-HA3), 1923 (Cse4-Myc6), and 2300 (H3-HA3, Cse4-Myc6) carrying the minichromosome was cross-linked with formaldehyde and immunoprecipitated with anti-HA or anti-Myc antibodies. DNA was eluted off the beads, resolved on a denaturing polyacrylamide gel and analyzed with a LNA probe for CDEI/II. B) BglII treated chromatin of the strains 1021 (wt), 1923 (Cse4-Myc6), and 2300 (H3-HA3, Cse4-Myc6) carrying the minichromosome was cross-linked with formaldehyde and immunoprecipitated with anti-Myc antibodies. Immunoprecipitated DNA was purified, size fractionated and subjected to qPCR analysis. C) Same as in (B) but performed with the native chromosome. The strains 2059 (wt), 2562 (Cse4-Myc6) and 2561 (Cse4-Myc6, H3-HA3) had CEN DNA of the native chromosome IV flanked with BglII.
(TIF)

Figure S6 H3 and Cse4 dimers can be covalently cross-linked via disulfide bonds between cysteine residues in the four-helix bundle. A) Structure of the four-helix bundle of the H3 homodimer, the CENP-A homodimer and the H3/CENP-A heterodimer. The yeast H3 histone fold domain is shown with alanine 111 and the human CENP-A histone fold domain with leucine 112 mutated to cysteines according the published nucleosome structures [41], [61]. The H3/CENP-A heterodimer is modeled by superimposition of the two published homodimer structures. Sulfur atoms are depicted in yellow. B) Cysteine-containing versions of recombinant yeast full-length H3 and the histone fold domain of Cse4 were expressed together and separately in bacteria. Crude bacterial lysates were separated on SDS-PAGE and analyzed by Western blot with Streptavidin-HRP recognizing histone H3 tagged with Avitag and anti-Penta-His antibody recognizing Cse4 tagged with His6. H3/H3 homodimers, Cse4/Cse4 homodimers and H3/Cse4 heterodimers are indicated.
(TIF)

Figure S7 Cysteine-containing versions of histone H3 but not Cse4 can be cross-linked on chromatin ex vivo. Chromatin pellets were treated with DTNB to facilitate the disulfide bond formation between the cysteine side chains. Proteins were then eluted with SDS-PAGE loading buffer without β-mercaptoethanol and separated on SDS-PAGE. Western blots were analyzed with anti-HA antibody recognizing tagged H3 (A) or anti-Myc antibody recognizing tagged Cse4 (B). The strains were 1021 (wt), 1266 (H3-HA3), 1268 (H3-HA3 (A111C)) 1924 (Cse4-Myc3 (L204C)), 1949 (Cse4-Myc3 (L204C) H3 (A111C)), 1953 (Cse4-Myc3 (L204C) H3-HA3 (A111C)), and 1955 (Cse4-Myc6 (L204C) H3-HA3 (A111C)).
(TIF)

Figure S8 ChIP efficiencies of core histones and Cse4 at different locations along a chromosome. Typical ChIP efficiencies are plotted according to the data in previous reports (see main text). The ChIP efficiency of histones and Cse4 at the centromere is usually reported to be in the range of 1% whereas DNA sequences from the chromosome arms are co-immunoprecipitated with the conventional histones with about 5–10 fold higher efficiency and with Cse4 with about 5–10 fold lower efficiency.
(TIF)

Table S1 List of yeast strains.
(DOC)
