# Supplementary figures and images for: Histone H3 Localizes to the Centromeric DNA in Budding Yeast
Source: PLoS Genet. 2012 May 31;8(5):e1002739. doi: 10.1371/journal.pgen.1002739 (PMC3364953; doi:10.1371/journal.pgen.1002739)

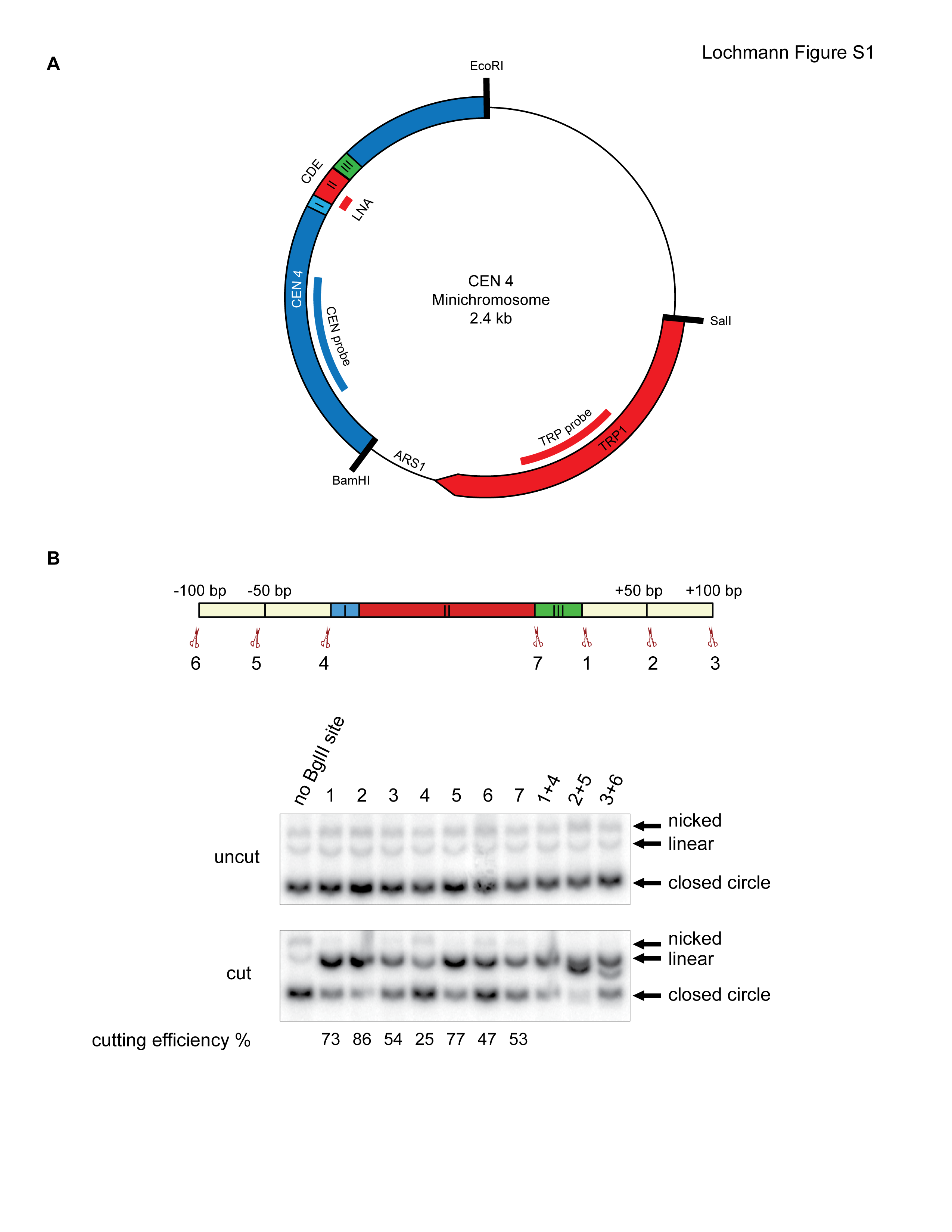

Supplement: Figure S1 — Accessibility of restriction endonuclease sites in the centromeric region of the minichromosome. A) Map of the minichromosome. The construct contains 850 bp of pericentromeric sequence of chromosome IV, TRP1 marker and ARS1. B) Top: Scheme of CEN4 with CDEI, CDEII and CDEIII indicated. The scissors indicate BglII sites in the different constructs. Bottom: The efficiency of a minichromosome digest at the indicated sites. DNA was isolated from BglII-treated lysates of strains carrying different minichromosomes, resolved on a 1% agarose gel and analyzed with a 32P labeled TRP1 probe. (TIF) [file pgen.1002739.s001.tif]

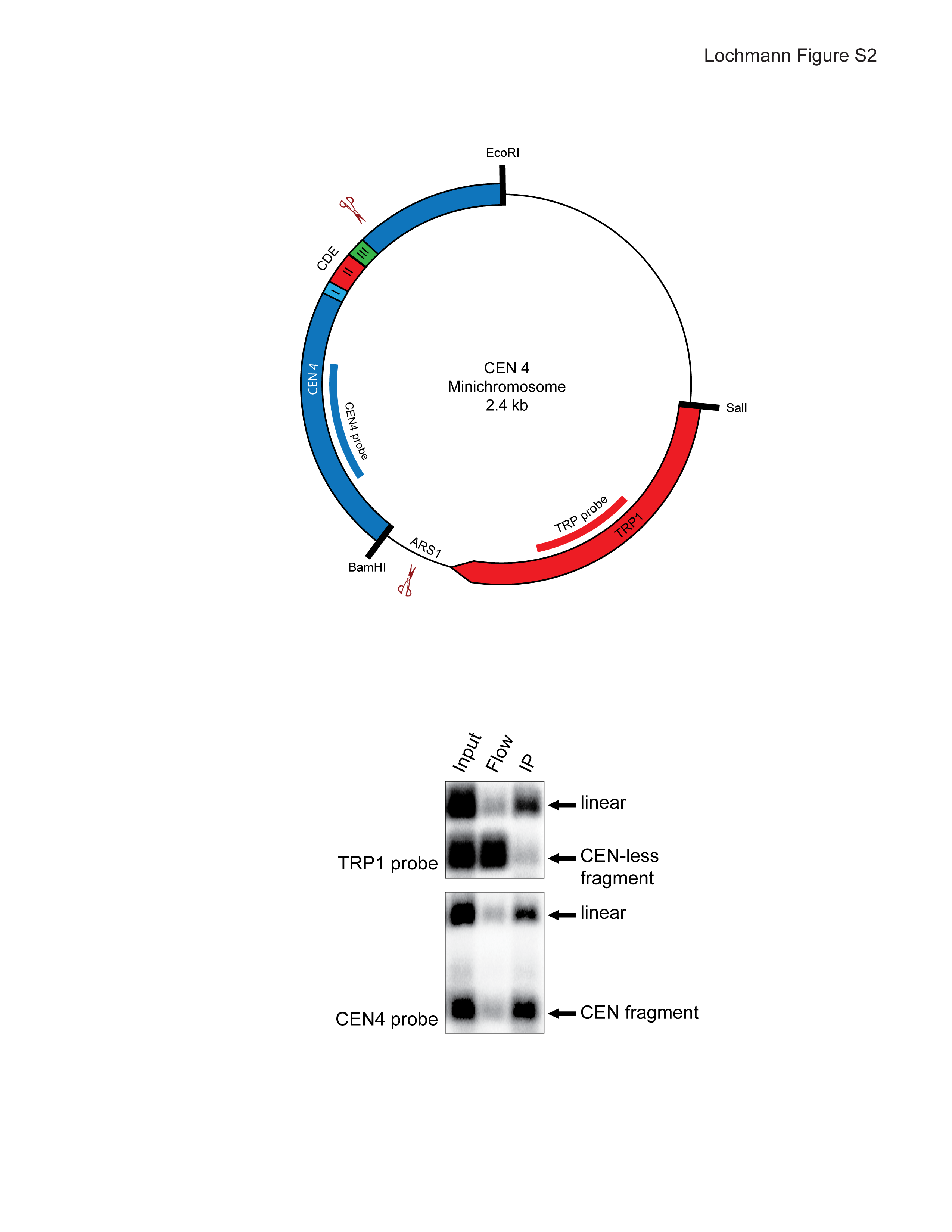

Supplement: Figure S2 — Cse4 nucleosome remains restricted to the CEN DNA in the course of immunoprecipitation procedure. Top: Map of the minichromosome utilized in the experiment. The construct contains 850 bp of pericentromeric sequence of chromosome IV, TRP1 marker and ARS1. BglII restriction sites are located 50 bp downstream of CDEIII and in ARS1 and are indicated with scissors. Bottom: BglII-treated chromatin of a strain 1498 (Cse4-HA6) carrying the minichromomosome was immunoprecipitated with anti-HA antibody without cross-linking. A long version of the procedure with 2 hours restriction digest was used. The DNA was eluted off the beads, purified via phenol/chloroform extraction and ethanol precipitation and separated on a 1% agarose gel. Southern blot was analyzed with a TRP1 probe to detect CEN-less fragment and a CEN4 probe hybridizing to the pericentromeric sequence to detect a fragment of the minichromosome containing CEN4. (TIF) [file pgen.1002739.s002.tif]

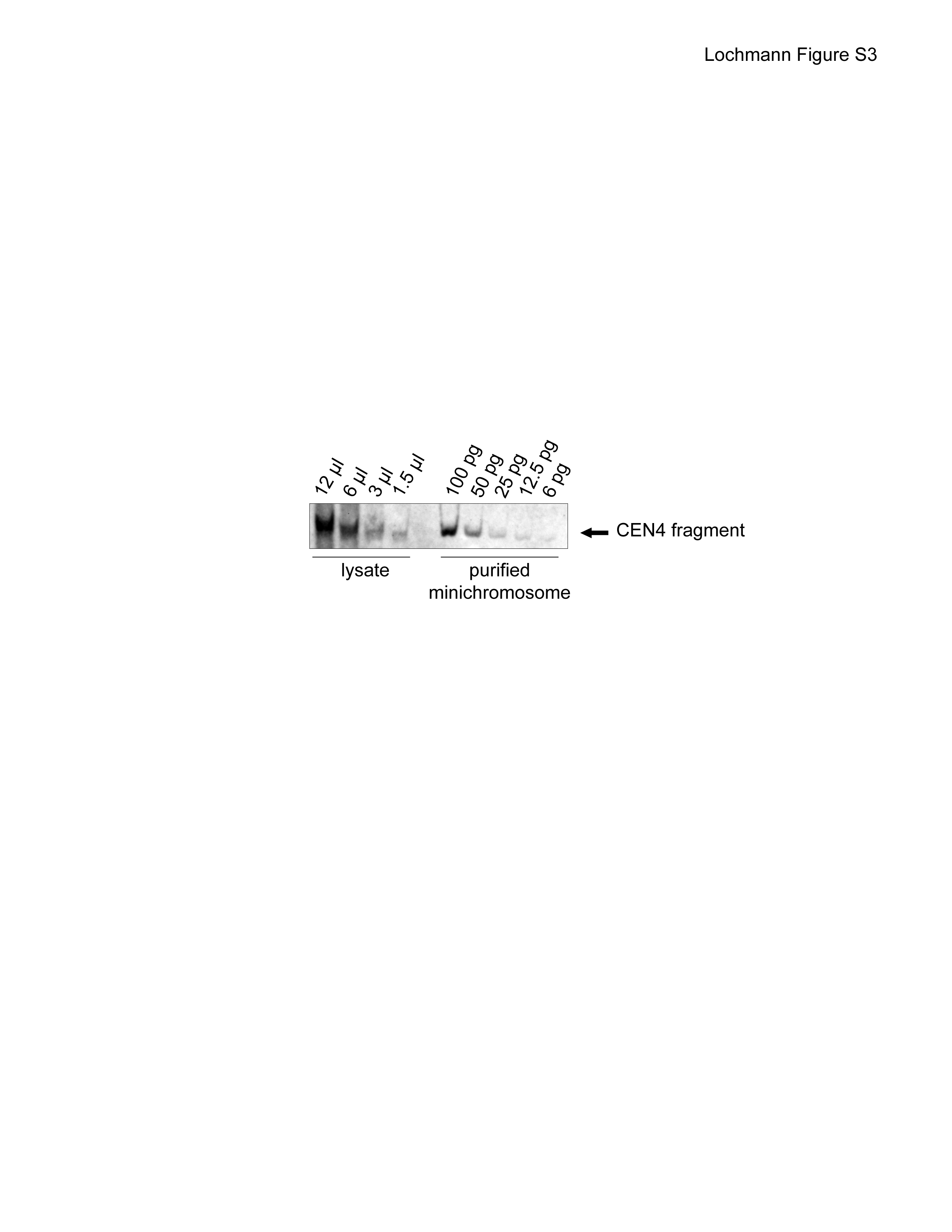

Supplement: Figure S3 — Sensitivity of the Southern blot detection with double DIG-labeled LNA probe for CDEI/II. DNA purified from BglII-treated lysate of a strain 1021 carrying the minichromosome with BglII restriction sites 50 bp upstream and downstream of CEN4 and known quantities of the minichromosome purified from bacteria (miniprep) and digested with BglII were resolved on a 6% denaturing TBE polyacrylamide gel and analyzed by Southern blot with the LNA probe for CDEI/II. (TIF) [file pgen.1002739.s003.tif]

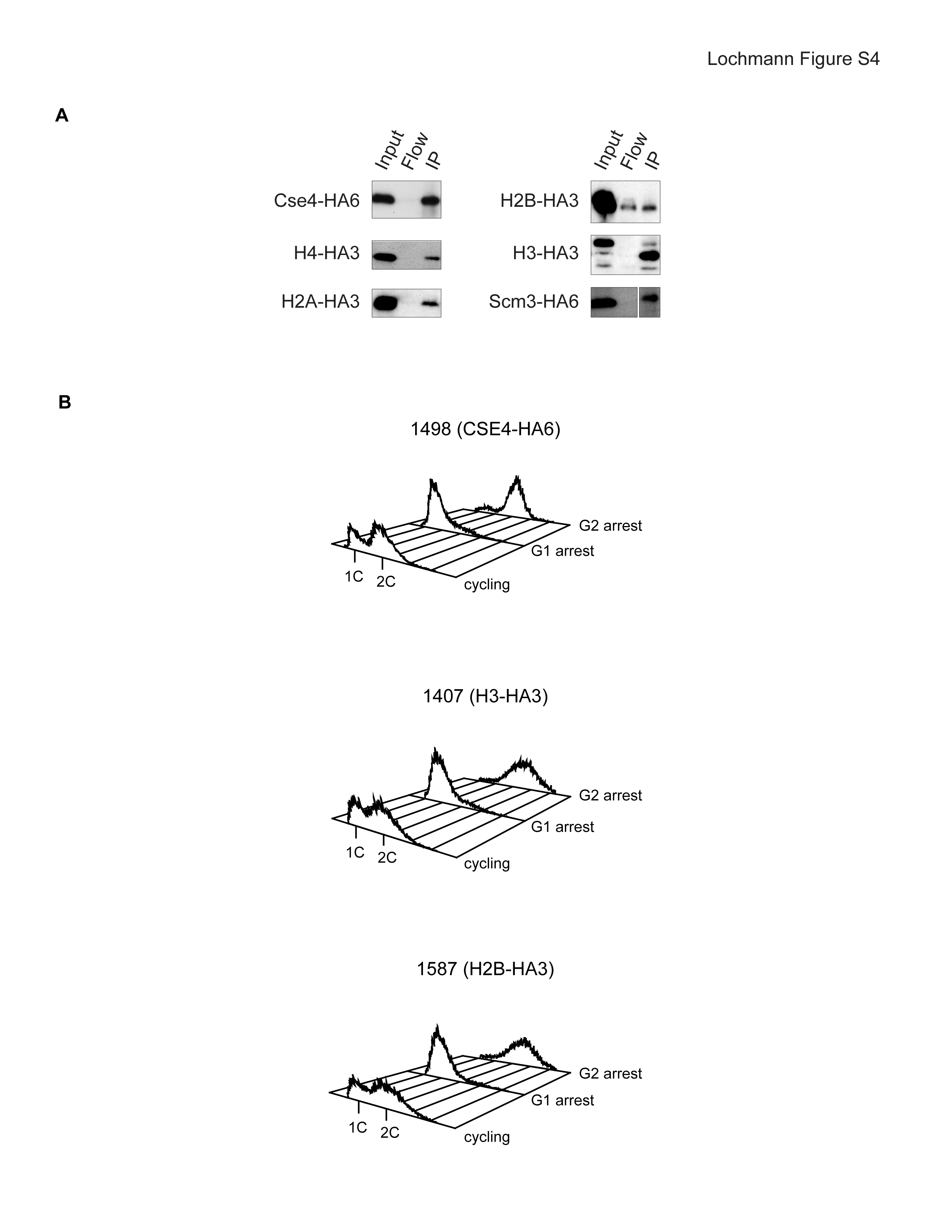

Supplement: Figure S4 — (A) Anti-HA Western blots of samples from ChIP experiments. Input, unbound fraction and eluted beads were separated on SDS-PAGE. (B) FACS analysis of the arrested yeast cultures in the experiment in Figure 2A. (TIF) [file pgen.1002739.s004.tif]

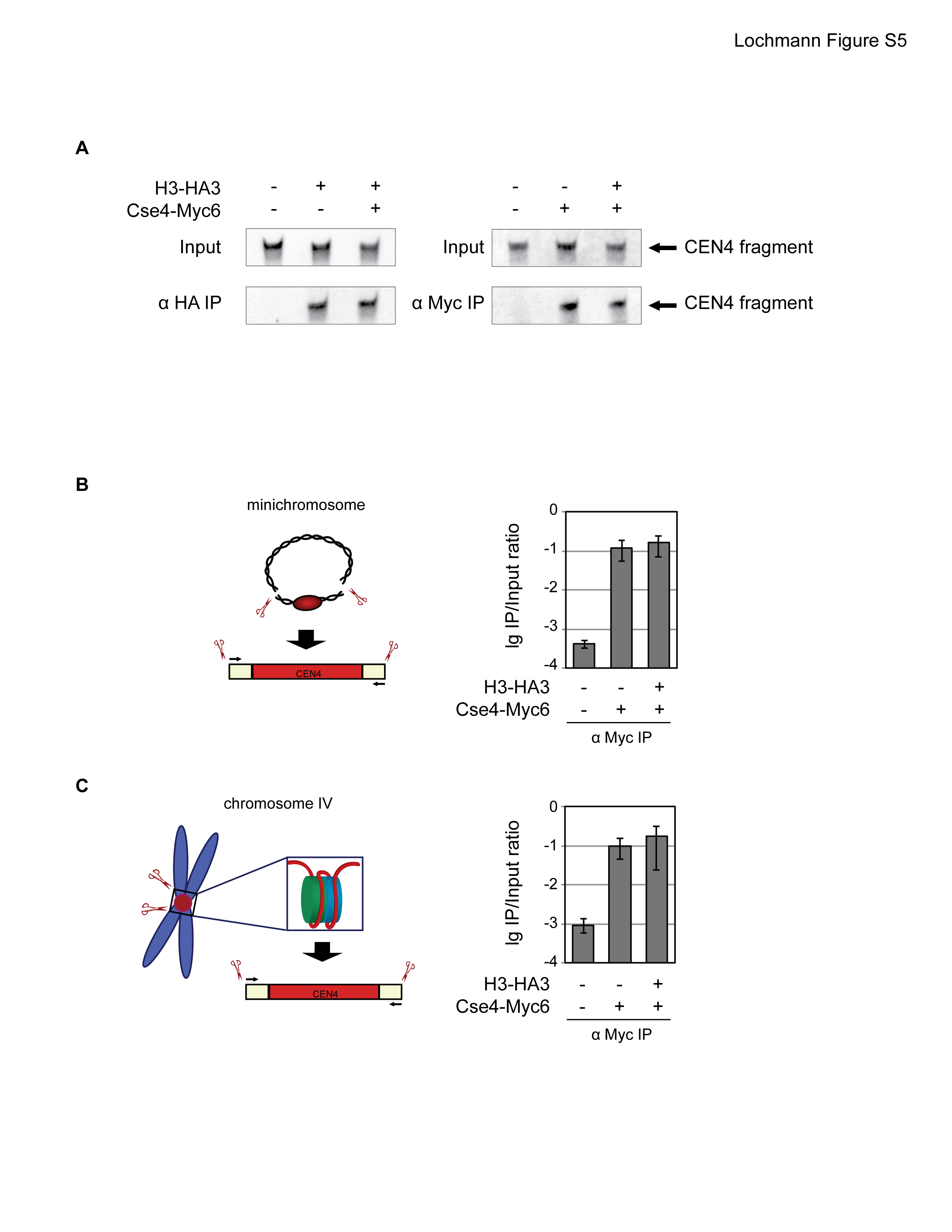

Supplement: Figure S5 — ChIP of minichromosomal and native CEN DNA fragment after formaldehyde cross-link. A) BglII-treated chromatin of the strains 1021 (wt), 1407 (H3-HA3), 1923 (Cse4-Myc6), and 2300 (H3-HA3, Cse4-Myc6) carrying the minichromosome was cross-linked with formaldehyde and immunoprecipitated with anti-HA or anti-Myc antibodies. DNA was eluted off the beads, resolved on a denaturing polyacrylamide gel and analyzed with a LNA probe for CDEI/II. B) BglII treated chromatin of the strains 1021 (wt), 1923 (Cse4-Myc6), and 2300 (H3-HA3, Cse4-Myc6) carrying the minichromosome was cross-linked with formaldehyde and immunoprecipitated with anti-Myc antibodies. Immunoprecipitated DNA was purified, size fractionated and subjected to qPCR analysis. C) Same as in (B) but performed with the native chromosome. The strains 2059 (wt), 2562 (Cse4-Myc6) and 2561 (Cse4-Myc6, H3-HA3) had CEN DNA of the native chromosome IV flanked with BglII. (TIF) [file pgen.1002739.s005.tif]

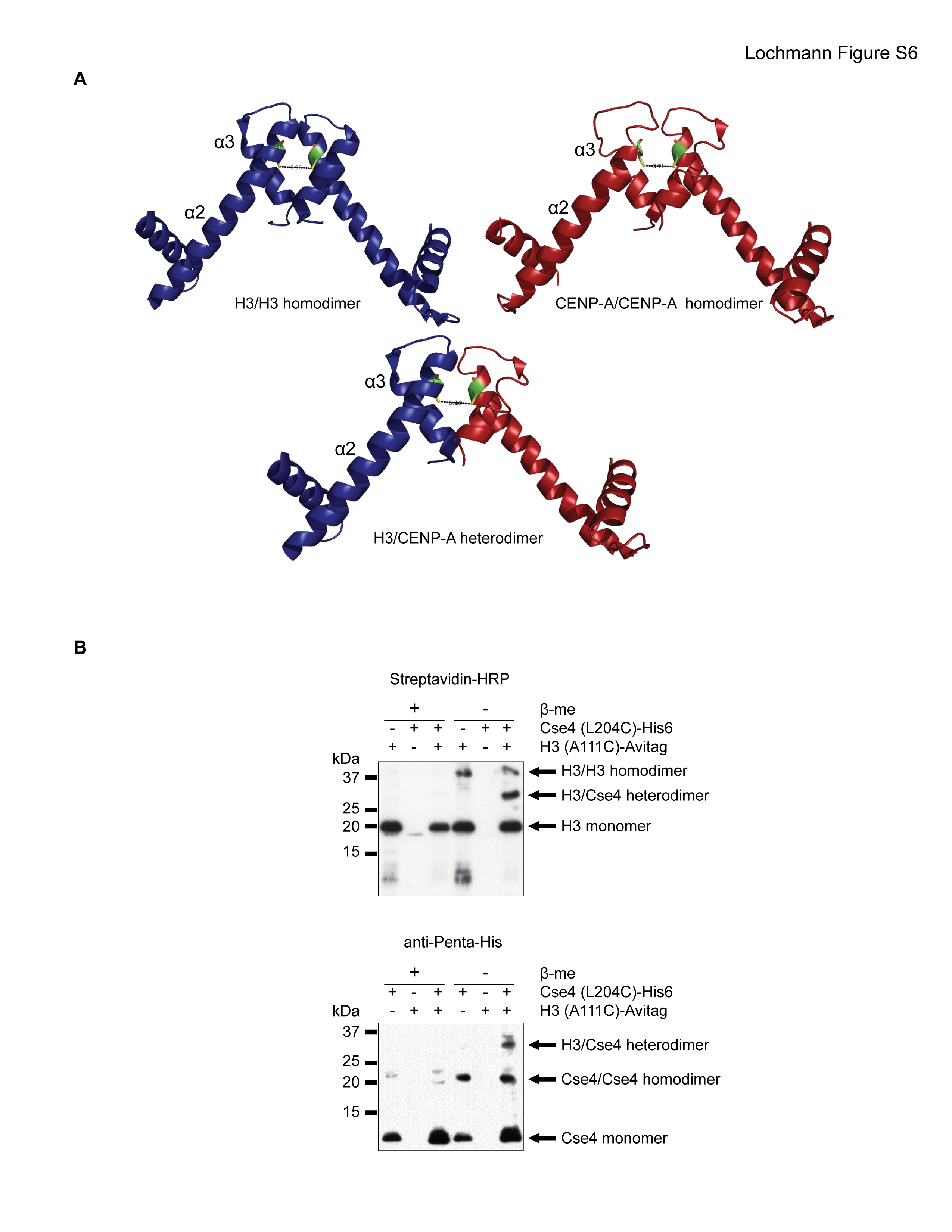

Supplement: Figure S6 — H3 and Cse4 dimers can be covalently cross-linked via disulfide bonds between cysteine residues in the four-helix bundle. A) Structure of the four-helix bundle of the H3 homodimer, the CENP-A homodimer and the H3/CENP-A heterodimer. The yeast H3 histone fold domain is shown with alanine 111 and the human CENP-A histone fold domain with leucine 112 mutated to cysteines according the published nucleosome structures [41], [61]. The H3/CENP-A heterodimer is modeled by superimposition of the two published homodimer structures. Sulfur atoms are depicted in yellow. B) Cysteine-containing versions of recombinant yeast full-length H3 and the histone fold domain of Cse4 were expressed together and separately in bacteria. Crude bacterial lysates were separated on SDS-PAGE and analyzed by Western blot with Streptavidin-HRP recognizing histone H3 tagged with Avitag and anti-Penta-His antibody recognizing Cse4 tagged with His6. H3/H3 homodimers, Cse4/Cse4 homodimers and H3/Cse4 heterodimers are indicated. (TIF) [file pgen.1002739.s006.tif]

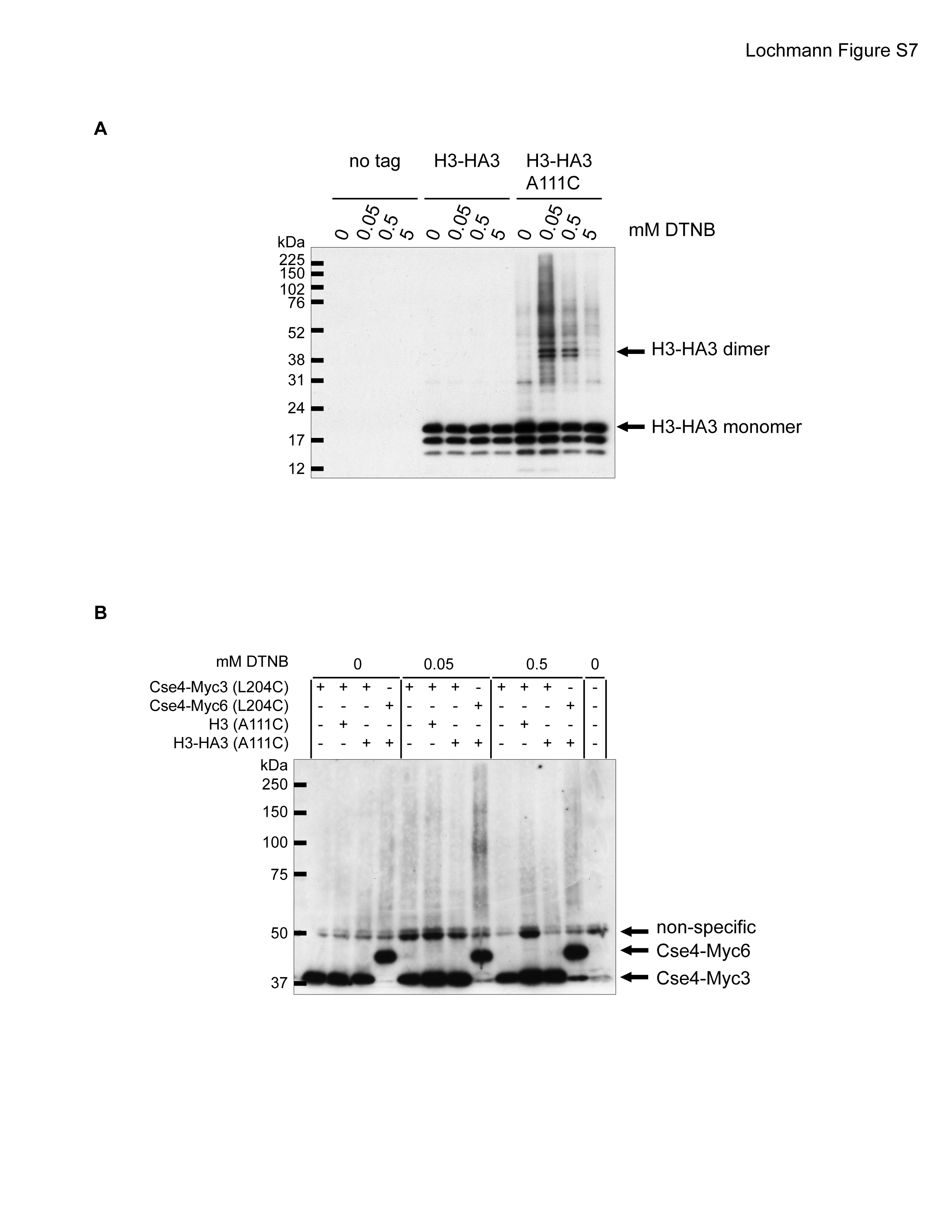

Supplement: Figure S7 — Cysteine-containing versions of histone H3 but not Cse4 can be cross-linked on chromatin ex vivo. Chromatin pellets were treated with DTNB to facilitate the disulfide bond formation between the cysteine side chains. Proteins were then eluted with SDS-PAGE loading buffer without β-mercaptoethanol and separated on SDS-PAGE. Western blots were analyzed with anti-HA antibody recognizing tagged H3 (A) or anti-Myc antibody recognizing tagged Cse4 (B). The strains were 1021 (wt), 1266 (H3-HA3), 1268 (H3-HA3 (A111C)) 1924 (Cse4-Myc3 (L204C)), 1949 (Cse4-Myc3 (L204C) H3 (A111C)), 1953 (Cse4-Myc3 (L204C) H3-HA3 (A111C)), and 1955 (Cse4-Myc6 (L204C) H3-HA3 (A111C)). (TIF) [file pgen.1002739.s007.tif]

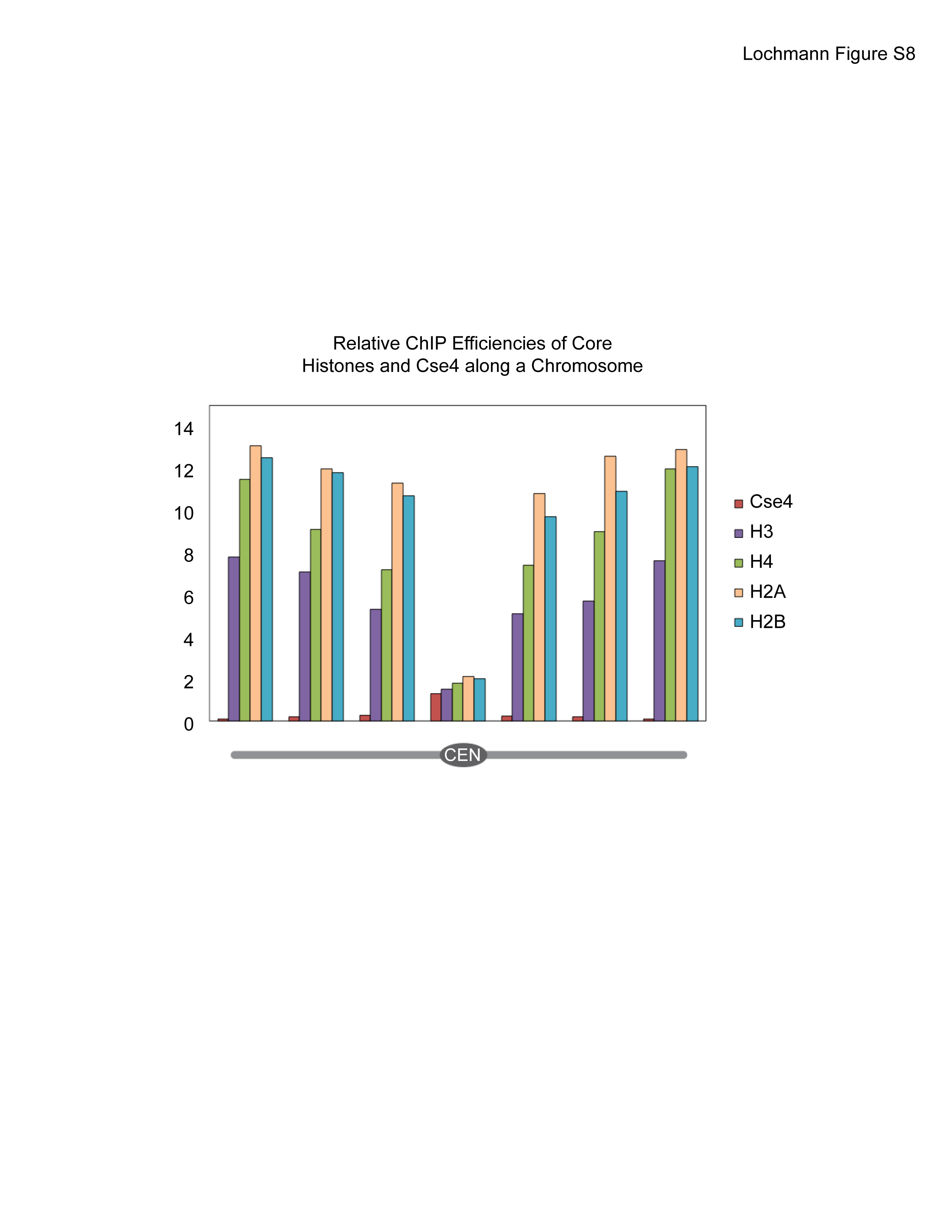

Supplement: Figure S8 — ChIP efficiencies of core histones and Cse4 at different locations along a chromosome. Typical ChIP efficiencies are plotted according to the data in previous reports (see main text). The ChIP efficiency of histones and Cse4 at the centromere is usually reported to be in the range of 1% whereas DNA sequences from the chromosome arms are co-immunoprecipitated with the conventional histones with about 5–10 fold higher efficiency and with Cse4 with about 5–10 fold lower efficiency. (TIF) [file pgen.1002739.s008.tif]
