# Supplementary material for: Histone H3 Localizes to the Centromeric DNA in Budding Yeast
Source: PLoS Genet. 2012 May 31;8(5):e1002739. doi: 10.1371/journal.pgen.1002739 (PMC3364953; doi:10.1371/journal.pgen.1002739)
Supplement: Table S1 — List of yeast strains. (DOC) [file pgen.1002739.s009.doc]

**Supplemental Table 1. List of yeast strains.**

|  | Genotype*^a^* |
| --- | --- |
| 1021 | Wild type |
| 1266 | *HHT1-HA3::HIS3* |
| 1268 | *hht2::HPH HHT1(A111C)-HA3::KAN::HIS3* |
| 1407 | *hht2::HPH HHT1-HA3::HIS3* |
| 1498 | *cse4::NAT CSE4-HA6::LEU2* |
| 1576 | *hta2::NAT HTA1-HA3::HIS3* |
| 1577 | *HHF1-HA3::HIS3* |
| 1587 | *htb2::NAT HTB1-HA3::HIS TetR-GFP-TAP::LEU2* |
| 1593 | *SCM3-HA6::HIS3* |
| 1923 | *MAT alpha* *cse4::NAT CSE4-Myc6::LEU2* |
| 1924 | *cse4::NAT CSE4(L204C)-Myc3::LEU2* |
| 1949 | *cse4::NAT CSE4(L204C)-Myc3::LEU2 hht2::HPH HHT1(A111C)::KAN* |
| 1953 | *cse4::NAT CSE4(L204C)-Myc3::LEU2 hht2::HPH HHT1(A111C)-HA3::KAN::HIS3* |
| 1955 | *cse4::NAT CSE4(L204C)-Myc6::LEU2 hht2::HPH HHT1(A111C)-HA3::KAN::HIS3* |
| 2042 | *hht2::HPH HHT1-HA3 CEN4* flanked with BglII +/-50 bp |
| 2043 | *cse4::NAT CSE4-HA6::LEU2 CEN4* flanked with BglII +/-50 bp |
| 2059 | *MAT alpha CEN4* flanked with BglII +/-50 bp |
| 2300 | *cse4::NAT CSE4-Myc6::LEU2 hht2::HPH HHT1-HA3::HIS3* |
| 2561 | *cse4::NAT CSE4-Myc6::LEU2 hht2::HPH HHT1-HA3::HIS3 CEN4* flanked with BglII +/-50 bp |
| 2562 | *cse4::NAT CSE4-Myc6::LEU2 CEN4* flanked with BglII +/-50 bp |

*^a^*All strains are isogenic in the W303 background and unless indicated otherwise have the genotype *MATa* *ade2-1 trp1-1 can1-100 leu2-3,112, his3-11,15 ura3* GAL psi^+^
